# Monitoring regulatory T cells in clinical samples: consensus on an essential marker set and gating strategy for regulatory T cell analysis by flow cytometry

**DOI:** 10.1007/s00262-015-1729-x

**Published:** 2015-06-28

**Authors:** Saskia J. A. M. Santegoets, Eveline M. Dijkgraaf, Alessandra Battaglia, Philipp Beckhove, Cedrik M. Britten, Awen Gallimore, Andrew Godkin, Cecile Gouttefangeas, Tanja D. de Gruijl, Hans J. P. M. Koenen, Alexander Scheffold, Ethan M. Shevach, Janet Staats, Kjetil Taskén, Theresa L. Whiteside, Judith R. Kroep, Marij J. P. Welters, Sjoerd H. van der Burg

**Affiliations:** 1 Department of Clinical Oncology, Leiden University Medical Center (LUMC), Leiden, The Netherlands; 2 Department of Obstetrics and Gynecology, Catholic University of Sacred Heart, Rome, Italy; 3 Division of Translational Immunology, Department of Surgical Oncology, German Cancer Research Center, Heidelberg University Hospital, Heidelberg, Germany; 4 TRON Translationale Onkologie an der Universitätsmedizin der Johannes Gutenberg-Universität Mainz GmbH, Mainz, Germany; 5 Present Address: GlaxoSmithKline, Cell Therapy Group, Immuno-Oncology & Combinations, Stevenage, UK; 6 Institute of Infection and Immunity, School of Medicine, Cardiff University, Cardiff, UK; 7 Department of Immunology, Institute for Cell Biology, Eberhard Karls University, Tübingen, Germany; 8 Department of Medical Oncology, Vrije Universiteit (VU) University Medical Center, Amsterdam, The Netherlands; 9 Laboratory of Medical Immunology, Department of Laboratory Medicine, Radboud University Medical Center, Nijmegen, The Netherlands; 10 Department of Cellular Immunology, Clinic for Rheumatology and Clinical Immunology, Charité - University Medicine, Berlin, Germany; 11 Laboratory of Immunology, National Institute of Allergy and Infectious Diseases, National Institutes of Health, Bethesda, MD USA; 12 Duke Center for AIDS Research, Duke University, Durham, NC USA; 13 Department of Surgery, Duke University Medical Center, Durham, NC USA; 14 Centre for Molecular Medicine Norway, Nordic EMBL Partnership, University of Oslo and Oslo University Hospital, Oslo, Norway; 15 K.G. Jebsen Centre for Cancer Immunotherapy and Biotechnology Centre, University of Oslo, Oslo, Norway; 16 Department of Pathology, University of Pittsburgh Cancer Institute, Pittsburgh, PA USA

**Keywords:** Consensus, Tregs, Monitoring, Flow cytometry, Phenotyping

## Abstract

Regulatory T cell (Treg)-mediated immunosuppression is considered a major obstacle for successful cancer immunotherapy. The association between clinical outcome and Tregs is being studied extensively in clinical trials, but unfortunately, no consensus has been reached about (a) the markers and (b) the gating strategy required to define human Tregs in this context, making it difficult to draw final conclusions. Therefore, we have organized an international workshop on the detection and functional testing of Tregs with leading experts in the field, and 40 participants discussing different analyses and the importance of different markers and context in which Tregs were analyzed. This resulted in a rationally composed ranking list of “Treg markers”. Subsequently, the proposed Treg markers were tested to get insight into the overlap/differences between the most frequently used Treg definitions and their utility for Treg detection in various human tissues. Here, we conclude that the CD3, CD4, CD25, CD127, and FoxP3 markers are the minimally required markers to define human Treg cells. Staining for Ki67 and CD45RA showed to provide additional information on the activation status of Tregs. The use of markers was validated in a series of PBMC from healthy donors and cancer patients, as well as in tumor-draining lymph nodes and freshly isolated tumors. In conclusion, we propose an essential marker set comprising antibodies to CD3, CD4, CD25, CD127, Foxp3, Ki67, and CD45RA and a corresponding robust gating strategy for the context-dependent analysis of Tregs by flow cytometry.

**Electronic supplementary material:**

The online version of this article (doi:10.1007/s00262-015-1729-x) contains supplementary material, which is available to authorized users.

## Introduction

Tregs play a key role in the regulation of self-tolerance and the maintenance of tissue homeostasis. Several human diseases such as autoimmune and immunodeficient conditions, chronic infections, and cancer have been associated with alterations in Treg numbers or function, and these alterations may contribute to disease progression and impact patient survival [1–3]. In cancer patients, it is well established that accumulation of Tregs is associated with tumor progression, poor prognosis, and the suppression of anti-tumor immune effector functions. Treg-mediated immunosuppression is therefore considered a major obstacle for successful cancer immunotherapy [4–6]. Given their potential to affect the outcome of immunotherapy trials, Tregs are being studied extensively in this context. The multitude of Treg definitions in the reported studies and the lack of functional Treg testing in immunomonitoring of clinical trials, however, make correct interpretation of data and comparisons between studies difficult, especially since knowledge of overlap between the identified Treg populations is missing and the methods to detect these cells differ per laboratory. As a result, blurred pictures emerge with respect to associations between clinical outcome and Tregs [7]. So far, Tregs have been identified through a number of different (combinations of) markers including CD4^pos^, Foxp3^pos/hi^, CD25^pos/hi^, CD127^neg/low^, CTLA-4^pos^, CD45RA^pos/neg^, Helios^pos^, CD39^pos^, and CD73^pos/neg^ using several different gating strategies [8–15]. The latter may form an important addition to misinterpretation of data sets since differences in gating strategies were found to be the biggest source for interassay variation in flow cytometry-based intracellular cytokine staining (ICS) assays [16, 17]. Similarly, a lack of adequate controls to guide the settings of gates may add another level of complexity to the analysis of Tregs.

To address these issues, the CIP organized a workshop on October 29, 2013 on the detection and functional testing of Tregs. This workshop, which hosted 40 researchers from seven countries in Europe and the USA, brought together leading experts in the field to (1) understand the state of the art of Treg research and to (2) define the most appropriate assays/markers to measure, quantify, and functionally assess Tregs within patient samples. As it became apparent during the workshop that a multitude of markers and combinations thereof is currently being used by the participants, a rationally composed ranking list of “Treg markers” was generated by the participants in the follow-up of the meeting. The preparation of this Treg marker list, subsequent data interpretation of the experiments performed at the LUMC, and subsequent discussions about and approval of the final conclusions were done through a series of circulating emails. Subsequently, the proposed Treg markers were tested in order to get insight into the overlap/differences between the most frequently used Treg definitions and their utility for Treg detection in various human tissues. This led to a context-dependent [i.e., peripheral blood/tumor/lymph node (LN)] essential marker set and robust gating strategy for the analysis of Tregs by flow cytometry.

## Materials and methods

### Cell samples

We acknowledge the concept of the minimal information about T cell assays (MIATA) reporting framework for human T cell assays [18]. Venous blood samples of healthy donors (HD) and recurrent ovarian cancer (OvCa) patients undergoing chemo-immunotherapeutic treatment (EM Dijkgraaf et al. submitted for publication) were drawn into sodium heparin collection tubes (Greiner Bio-one, Alphen a/d Rijn, the Netherlands) after signing informed consent. PBMCs were isolated using Ficoll (LUMC pharmacy, Leiden, the Netherlands) density gradient centrifugation, washed with PBS (B. Braun, Melsungen, Germany), cryopreserved in 90 % fetal calf serum (FCS; PAA Laboratories, Pasching, Austria) and 10 % DMSO (Sigma-Aldrich, St. Louis, MO, USA), and stored in the vapor phase of liquid nitrogen until further use [19]. TDLN and tumor samples were obtained from cervical cancer patients (CxCa) within the CIRCLE study after signed informed consent. The CIRCLE study investigates cellular immunity against HPV in HPV-induced (pre)malignant lesions and was approved by the Medical Ethical Committee of the LUMC [20]. Single-cell suspensions were prepared from TDLN and tumor samples using collagenase/DNase digestion or gentle MACS procedure, respectively. First, TDLN and tumor samples were cut into small pieces. Single-cell suspensions were prepared by incubating the TDLN pieces with 250 U/ml collagenase D (Roche, Almere, the Netherland) and 50 µg/ml DNase I (Roche) for 1 h at 37 °C, after which the TDLN was put through a cell strainer [21]. Single-cell suspensions of tumor samples were prepared by incubating the tumor pieces for half an hour at 37 °C in IMDM/10 % human AB serum (Greiner) supplemented with 50 µg/ml gentamycin (Life technologies, Bleiswijk, the Netherlands), 25 µg/ml Fungizone (Life Technologies), 10 % penicillin/streptomycin (Sigma), 1 mg/ml collagenase D, and 50 µg/ml DNAse I (dissociation mix), followed by gentleMACS dissociation procedure according to the manufacturers’ instructions. Next, cells were frozen and stored as above. The handling and storage of the PBMC, TDLN, and tumor samples were done according to the standard operation procedures (SOP) of the department of Clinical Oncology at the LUMC by trained personnel. The use of the above-mentioned patient materials was approved by the Medical Ethics Committee Leiden in agreement with the Dutch law for medical research involving humans.

### Treg enumeration by flow cytometry

The cryopreserved cell samples were thawed according to SOPs and as described before [19], and Treg subsets were assessed by flow cytometry staining. To this end, one million PBMCs or ~250,000–750,000 TDLN or tumor sample cells was used per condition. Since it has been described that Foxp3 staining can be highly variable and depend on the choice of antibody (clone), buffer, and/or fluorochrome [22–24] and the performance of a specific antibody is optimized by the manufacturer using their own permeabilization procedures, optimal Foxp3 staining was determined first. We selected four different Foxp3 antibodies on the basis of in-house availability, compatibility with the rest of our panel and with the LSR Fortessa optical configuration, and two different intranuclear staining kits. Optimal staining was determined by the analysis of the percentage of positive cells and at the strength of the positive signal (compared to the negative fluorescence minus one (FMO) signal). Antibodies and intranuclear staining kits used for Foxp3 staining setup were AF700-labeled Foxp3 (clone PCH101, eBiosciences), PE-labeled Foxp3 (clone PCH101, eBiosciences, and clone 206D, R&D systems), PE-CF594-labeled Foxp3 (clone 259D/C7, BD), AmCyan-labeled CD3 (clone SK7, BD), V500-labeled CD3 (clone UCHT1, BD), PE-CF594- or AF700-labeled CD4 (both clone RPA-T4, BD), PE-CY7-labeled CD25 (clone 2A3, BD), BV650-labeled CD127 (clone HIL-7R-M21, BD), the Foxp3/transcription factor staining buffer set (eBiosciences), and the BD Pharmingen Transcription Factor Buffer set (BD). Cell surface antibody staining was performed in PBS/0.5 % BSA/0.02 % sodium azide (PBA) buffer for 30 min at 4 °C. Intranuclear Foxp3 staining was conducted with the BD or eBiosciences Transcription Factor Buffer sets according to the manufacturers’ protocol. Analysis revealed that Foxp3 could be detected with all used clones when using the eBiosciences kit. Yet, staining intensity (and thus discrimination between negative and positive) was lower with the PCH101 clones when compared with the 206D (PE) clone (Supplementary figure 1a–c), which may be due to fluorochrome choice. Staining pattern and positive-to-negative signal ratio [i.e., staining index (SI)] of the 259D/C7 (PE-CF594) clone were most optimal with the BD TF kit (not shown) and were comparable to the staining pattern of the 206D clone using this kit, indicating that both antibodies could be used in our Treg panel (Supplementary figure 1d–f). After selection of the best Foxp3 antibody and intranuclear staining buffer set, all additional antibodies in the final panel were titrated, and spillover profiles were generated to ascertain that there was no spectral overlap of the selected antibodies into the secondary detectors. Optimal antibody concentrations were determined based on the following criteria: (a) frequency and (b) highest SI (positive mean divided by negative mean), and spillover profiles were generated as described by Murdoch et al. [25]. Antibodies and kits used in the final panel were V500-labeled CD3 (clone UCHT1, BD), AF700-labeled CD4 (clone RPA-T4, BD), PE-CY7-labeled CD25 (clone 2A3, BD), BV650-labeled CD127 (clone HIL-7R-M21, BD), APC-H7-labeled CD45RA (clone HI100, BD), PerCP-Cy5.5-labeled CD8 (clone SK1, BD), PE-CF594-labeled Foxp3 (clone 259D/C7, BD), BV421-labeled CTLA-4 (clone BNI3, BD), FITC-labeled Ki67 (clone 20Raj1, eBiosciences), APC-labeled Helios (clone 22F6, Biolegend), PE-labeled CD39 (clone ebioA1, eBiosciences), LIVE-DEAD^®^ Fixable yellow dead cell stain kit (Q-dot585, Life technologies), and the BD Pharmingen Transcription Factor Buffer set. Stained cells were acquired on a LSR Fortessa (BD) and analyzed using DIVA software version 6.2. Events collected were generally >200,000 per sample, except for one tumor-infiltrating lymphocyte (TIL) sample (~35,000 cells). In the latter, still adequate numbers (~400) of Tregs could be detected.

### Treg definitions and gating strategies

Tregs were analyzed according to three commonly used Treg definitions in the literature: (1) the CD25^pos^CD127^low^Foxp3^pos^ subset [definition 1 (def.1)] [9, 10], (2) the Foxp3^pos^Helios^pos^ Treg subset (def.2) [12, 26], and (3) the Foxp3^hi^CD45RA^neg^ activated Treg (aTreg) and Foxp3^int^CD45RA^pos^ naïve Treg (nTreg) subsets (def.3) [8, 11]. Gating for CD25 and CD127 (def.1), Foxp3 and Helios (def.2), and Foxp3 and CD45RA (def.3) Tregs was done on CD3^pos^CD4^neg^ (i.e., CD8^pos^) T cells and CD3^neg^ lymphocytes, respectively, and subsequently applied to CD3^pos^CD4^pos^ T cells (see also supplementary figure 2a, 3a, and 5a). Percentage of def.1, def.2, or def.3 Tregs is given as percentage within the CD4^pos^ population.

### Statistical analysis

Nonparametric (Wilcoxon signed-rank or Mann–Whitney test for two samples and Friedman or Kruskal–Wallis with Dunn’s multiple comparison test for multiple samples) and parametric (paired or unpaired *t* test for two samples or RM one-way ANOVA or ordinary one-way ANOVA with Tukey’s multiple comparison test for multiple samples) tests were performed as appropriate. All statistical tests were performed at the 0.05 significance level, and 95 % confidence intervals were two-sided intervals. For survival analysis, the OvCa patients undergoing chemo-immunotherapeutic therapy were grouped into two groups according to the median (i.e., grouped into below or above the median of the total group for each parameter), after which survival was tested using Kaplan–Meier method, and statistical significance of the survival distribution was analyzed by log-rank testing. Statistical analyses were performed using SPSS for Windows version 20.0 (IBM, USA) and GraphPad Prism 6.02 (San Diego, USA).

## Results

### Generation of a rationally ranked Treg marker list

During the CIP workshop, a number of Treg analysis methods were presented. These analyses were discussed, a number of questions were formulated, and during the follow-up of the meeting, a rationally composed ranking list of “Treg markers” was generated. All markers suggested, and the rationale to use them is given in Table 1. To test these markers and get insight into the overlap/differences between the most frequently used human Treg definitions, we included markers 1–8, 10, and 11 for direct ex vivo analysis of peripheral blood samples from six HD and OvCa patients, and LN and tumor samples obtained from CxCa patients. Markers were included based on the number of participants opting for inclusion of the marker and/or their known association with Tregs. LAP/GARP (number 9) was excluded as this marker is only expressed >24 h following in vitro activation.

**Table 1:** Treg marker list generated after inquiry among workshop participants

Order^a^	Marker	Expression	Location	Comment	# of responding participants^b^	References
1	CD3	Directly ex vivo	Cell surface	Essential	All	
2	CD4	Directly ex vivo	Cell surface	Essential	All	
3	CD25	Directly ex vivo	Cell surface	Essential	All	
4	Foxp3	Directly ex vivo	Intranuclear	Essential	All	
5	CD127	Directly ex vivo	Cell surface	Essential; Low/absent on Tregs	All	[9, 10]
6	CD45RA	Directly ex vivo	Cell surface	Discriminates between naïve and TCR-triggered Tregs	All	[8, 11]
7	Helios	Directly ex vivo	Intranuclear	Used to discriminate between Foxp3^pos^ Tregs (Helios^pos^) and Foxp3^pos^ activated T cell (Helios^neg^); superior to CD25 and CD127 in activated conditions (such as systemic lupus erythematosus (SLE))	7/10	[12, 13, 47, 48]
8	Ki67	Directly ex vivo	Intranuclear	Identifies recently activated/proliferating Tregs	7/10	[36, 37]
9	LAP/GARP	Upon activation in PBMC/directly ex vivo on TIL	Cell surface	Expressed by human Tregs upon in vitro activation (>24 h); low/absent on ex vivo PBMC-derived Tregs, high expressed on ex vivo TIL-derived CD4^pos^ T cells	5/10	[42–45]
10	CTLA4	Directly ex vivo	Intracellular	Co-expressed with Foxp3 at higher levels than in activated conventional T cells	3/10	[38]
11	CD39	Directly ex vivo	Cell surface	Present on suppressive iTregs and nTregs	2/10	[14, 15]
12	CD73	Directly ex vivo	Intracellular	Expressed on iTregs; absent on nTregs	2/10	[14, 15]
14	CD147	Directly ex vivo	Cell surface	Identifies activated and highly suppressive Tregs	1/10	[36, 40, 41]
13	CD137^pos^/CD154^neg^	upon activation (>6 h)	Cell surface	Discriminates activated Tregs from activated CD137^pos^CD154^pos^ Tconv	1/10	[49, 50]
14	CD49d	Directly ex vivo	Cell surface	Activation marker, Tregs are CD49d^neg^	1/10	[51]
15	IL-2	Upon activation (>6 h) with PMA/ion.	Intracellular	Discriminates between Foxp3^pos^IL-2^neg^ Treg and Foxp3^pos^IL-2^pos^ non-Tregs	1/10	[12, 13, 26]
16	Neuropilin-1	Directly ex vivo	Cell surface	Expressed on a highly efficient Treg subset (CD25^pos^Foxp3^pos^) in TDLN	1/10	[52–54]
17	CCR2	Upon activation	Cell surface	Expressed on activated, tumor antigen-specific T cells in cancer patients (bone marrow and peripheral blood)	1/10	Beckhove et al. unpubl. data

^a^Order based on the number of participants who would like to add the indicated marker

^b^Number of participants (of total) who opted for inclusion of the indicated marker. From 10 participants we received a response to our request in writing to generate a marker list indicating what markers they would like to add, in what order and context (tissue or site specific?) and why

### Analysis of Tregs according to commonly used Treg definitions

Tregs were analyzed according to three commonly used Treg definitions in the literature [8–12, 26].

#### Definition 1: CD25^pos^CD127^low^Foxp3^pos^ Tregs

**Fig. 1:**
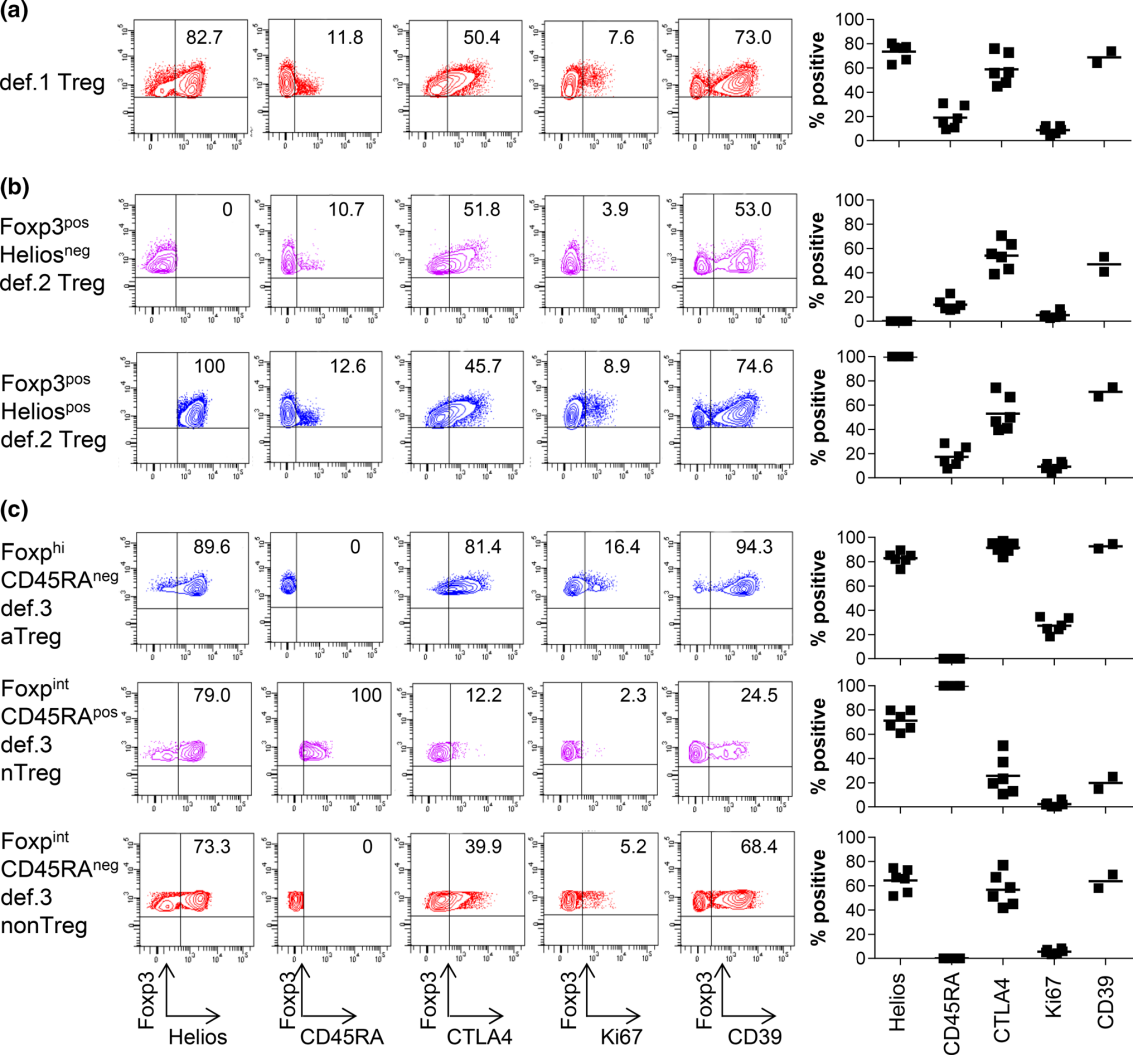
CD25^pos^CD127^low^Foxp3^pos^ def.1, Foxp3^pos^Helios^pos^ def.2, and Foxp3^hi^CD45RA^neg^ def.3 aTregs express high levels of Treg-associated markers, suggesting that they are bona fide Tregs. Phenotypic characterization of def.1, def.2, and def.3 Tregs was performed by flow cytometry. Gating of the three different Treg definitions was performed as described in supplementary figs. 2a, 3a, and 5a. Expression of the Treg-associated markers Helios, CD45RA, CTLA4, Ki67, and CD39 is depicted for a representative healthy donor (HD; *left*) and multiple HD (*right*; Helios/CD45RA/CTLA4/Ki67 for six and CD39 for two HD) for **a** CD25^pos^CD127^low^Foxp3^pos^ def.1 Tregs, **b** Foxp3^pos^Helios^neg^ and Foxp3^pos^Helios^pos^ def.2 Tregs, and **c** Foxp3^hi^CD45RA^neg^ def.3 aTregs, Foxp3^int^CD45RA^pos^ def.3 nTregs, and Foxp3^int^CD45RA^neg^ def.3 non-Tregs. Percentage Helios/CD45RA/CTLA4/Ki67/CD39 expression is given as percentage of the designated population in the *upper right* quadrant in the FACS plot for the representative HD (*left*) and as mean percentage for the six HD (*right*)

Figure 1a shows the expression of the different markers in def.1 Tregs. The gating strategy for the CD25^pos^CD127^low^Foxp3^pos^ def.1 Treg subset is given for a representative HD in supplementary Fig. 2a. Cells expressing Foxp3 comprised 78.7 % (range 70.5–85.1 %) of the CD25^pos^CD127^low^ subpopulation. Due to variability in CD127 expression (Supplementary figure 2b, c), enumerating def.1 Tregs solely based on CD25 and CD127 is highly variable between HD and most likely leads to an overestimation of the number of Tregs (mean 17.6 %, range 7.2–30.4 %). Inclusion of Foxp3 resulted in less variation in the percentage of def.1 Tregs (mean 6.9 %, range 4.6–8.8 %) as would be expected among a group of HD, suggesting that simultaneous staining with CD25, CD127, and Foxp3 is needed for reliable measurement of def.1 Tregs. Further characterization of the CD25^pos^CD127^low^Foxp3^pos^ subset revealed that 75 % of these cells were Helios positive (Fig. 1a). Moreover, the majority of CTLA-4 and Ki67 expressing CD4^pos^ T cells were found in the CD25^pos^CD127^low^Foxp3^pos^ population (data not shown). These observations add to the notion that bona fide Tregs are detected when the CD25^pos^CD127^low^Foxp3^pos^ def.1 subset definition for Treg enumeration is used.

**Fig. 2:**
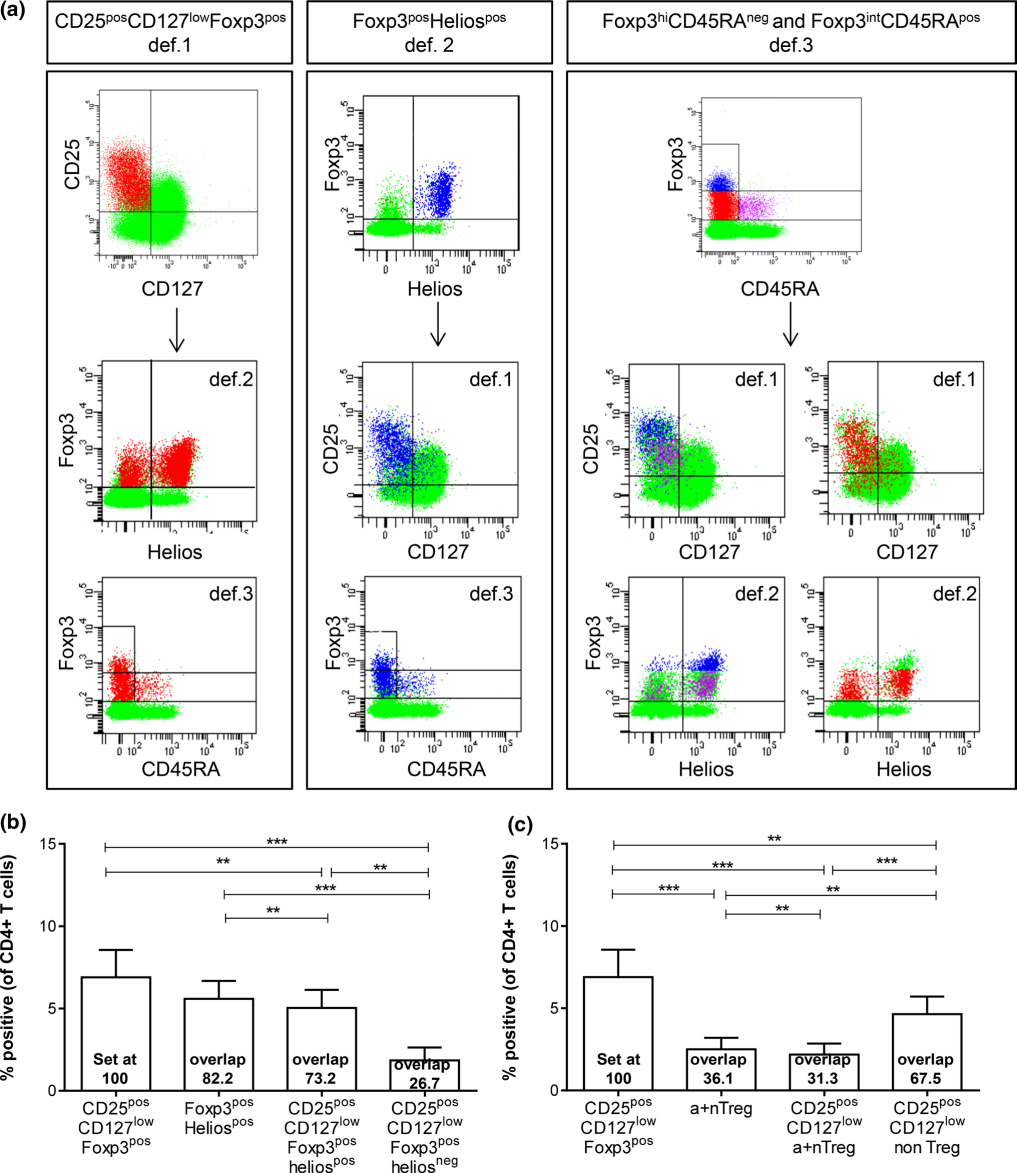
Treg enumeration based solely on Foxp3 and Helios (def.2) or Foxp3 and CD45RA (def.3) led to an underestimation of CD25^pos^CD127^low^Foxp3^pos^ def.1 Tregs through exclusion of def.1 Treg cells in the Foxp3^pos^Helios^neg^ (def.2) or Foxp3^int^CD45RA^neg^ non-Treg (def.3) populations. Overlap between the three most commonly used Treg definitions (def.1, def.2, and def.3) is given for **a** a representative HD and **b,** **c** six HDs. **a** Distribution of def.1 Tregs is shown in def.2 and def.3 populations (*left*), of def.2 Tregs is shown in def.1 and def.3 populations (*middle*), and of def.3 Tregs is shown in def.1 and def.2 populations (*right*). Percentage of Tregs analyzed via def.1, def.2, or a combination thereof **b** and via def.1, def.3, or a combination thereof **c** is depicted as percentage of CD4^pos^ T cells. Overlap between the designated populations is calculated in relation to def.1 Tregs (set at 100) and given in the *bar graph* for each population

#### Definition 2: Foxp3^pos^Helios^pos^ Tregs

The gating strategy for the Foxp3^pos^Helios^pos^ def.2 Treg subset is given for a representative HD in supplementary figure 3a. Analysis revealed that 5.6 % of CD4^pos^ T cells is Foxp3^pos^Helios^pos^ (range 4.1–7.1 %), and Foxp3^pos^Helios^neg^ cells accounted for 2.9 % (range 1.9–4.4 %) of CD4^pos^ T cells. Interestingly, Foxp3 expression of Foxp3^pos^Helios^neg^ cells was significantly lower than that of Foxp3^pos^Helios^pos^ cells (Supplementary figure 3b, c). Further characterization of the def.2 Treg subsets revealed that the majority of the Foxp3^pos^Helios^pos^ cells (mean 88 %, range 84.7–90.7 %) were found inside the CD25^pos^CD127^low^ def.1 Treg subset (Supplementary figure 4). Moreover, 64 % of Foxp3^pos^Helios^neg^ cells (range 52.0–72.9 %) could also found within that CD25^pos^CD127^low^ gate. Expression levels of CTLA4 and CD45RA were found similar in Foxp3^pos^Helios^neg^ and Foxp3^pos^Helios^pos^ cells (Fig. 1b). Together, this indicates that although probably polluted with Foxp3^pos^ activated effector T cells, the population of Foxp3^pos^Helios^neg^ cells also contain considerable amounts of Tregs according to definition 1. Interestingly, Foxp3^pos^Helios^pos^ cells expressed significantly more Ki67 compared with Foxp3^pos^Helios^neg^ cells, suggesting that Foxp3^pos^Helios^pos^ cells, which also express higher levels of Foxp3, represent more recently activated Tregs (*p* = 0.03; Fig. 1b).

#### Definition 3: Foxp3^hi^CD45RA^neg^ a Treg and Foxp3^int^CD45RA^pos^ n Tregs

The gating strategy for the Foxp3^hi^CD45RA^neg^ and Foxp3^int^CD45RA^pos^ def.3 Treg subsets is given for a representative HD in supplementary figure 5a. Foxp3^hi^CD45RA^neg^ aTreg accounted for 1.1 % (range 0.8–1.6 %) and Foxp3^int^CD45RA^pos^ nTreg for 1.4 % (range 0.6–2.5 %) of CD4^pos^ T cells. Remarkably, the so-called Foxp3^int^CD45RA^neg^ non-Treg subset accounted for 6.0 % (range 4.2–7.4 %) of CD4^pos^ T cells, and this was significantly more than the aTreg and nTreg frequencies detected (*p* < 0.001) (Supplementary figure 5b). Further characterization revealed that the majority of aTregs and nTregs could be found within the CD25^pos^CD127^low^ def.1 and Helios^pos^ def.2 Treg populations. Yet, the so-called non-Treg population also comprised considerable numbers of the def.1 (77.3 %) and def.2 (56.2 %) Tregs, indicating that the def.3 non-Treg population still contained high numbers of Tregs according to the other definitions (Supplementary figure 6a, b, d). Moreover, the frequency of def.1 or def.2 Tregs within the non-Treg population was significantly higher than within the aTreg and nTreg populations (Supplementary figure 6a, c, e). As expected, the aTreg, but not the nTreg population, displayed an activated profile indicated by high levels of Ki67 and CTLA4 expressions (% and mean fluorescence intensity; Fig. 1c and data not shown, respectively) sustaining the notion that this preset profile is likely to accurately detect activated Tregs.

#### Expression of ectonucleoside triphosphate diphosphohydrolase-1 (CD39)

It has been described that human Tregs express CD39, an ectonucleotidase involved in adenosine triphosphate (ATP) breakdown and the production of immunosuppressive adenosine, thereby suggesting that CD39 may be a functional marker on Tregs [27, 28]. To study the expression of CD39 in relation to the three commonly used Treg definitions, CD39 was included in our flow cytometric marker panel, and two HD-derived PBMC samples were analyzed. Although the majority of CD39 can be found in CD25^pos^CD127^low^Foxp3^pos^ def.1 Tregs (~70 %), expression of CD39 is not def.1 Treg exclusive (Supplementary figure 7a, d). Similar results were found for Foxp3^pos^Helios^pos^ def.2 Treg and the Foxp3CD45RA def.3 Treg subsets (Supplementary figures 7b, c, e, f). Interestingly and indeed suggestive of their functional potential, CD39 expression is much higher in Foxp3^hi^CD45RA^neg^ aTregs than in Foxp3^int^CD45RA^pos^ nTregs. Thus, CD39 expression seems to be present especially on activated Tregs, but its expression is not Treg exclusive. Within the activated Treg populations, it identifies the same population of cells, that is, CD45RA^neg^ and CTLA4^pos^. CD39 expression therefore falls into the category of markers for identifying the activated subset of Tregs. Of note, it has been demonstrated that CD39, when combined with CD25, can be used to identify and isolate Tregs with strong suppressive activity [29, 30]. Gating on the cell surface markers CD25^pos^, CD127^low^, and CD39^pos^ yielded 75–80 % Foxp3^pos^ cells in our hands (Supplementary figures 7 and 8).

Based on the expression of high levels of CD25, Helios, CTLA-4, and CD39, the CD25^pos^CD127^low^Foxp3^pos^ def.1, Foxp3^pos^Helios^pos^ def.2, and Foxp3^hi^CD45RA^neg^ def.3 T cells were classified as bona fide Tregs.

### Overlap between the three Treg definitions

Next, the overlap between the def.1, def.2, and def.3 Tregs was determined (Fig. 2a–c). As expected, there was considerable overlap between the three Treg definitions. The overlap between the CD25^pos^CD127^low^Foxp3^pos^ def.1 Tregs and the Foxp3^pos^Helios^pos^ def.2 Tregs is approximately 73 %, and thus, Treg enumeration based solely on Foxp3 and Helios may lead to an underestimation in Tregs of ~27 % through exclusion of CD25^pos^CD127^low^Foxp3^pos^ cells in the Foxp3^pos^Helios^neg^ population (range 20.2–35.3 % of CD25^pos^CD127^low^Foxp3^pos^ Tregs; supplementary figure  4c). Furthermore, Treg measurements based solely on Foxp3 and CD45RA (def.3) led to an underestimation of the number of def.1 Tregs of 67.5 % through exclusion of the so-called Foxp3^int^CD45RA^neg^ non-Tregs (range 61.0–73.5 % of CD25^pos^CD127^low^Foxp3^pos^ Tregs; supplementary figure 6c).

### Treg enumeration in PBMC, TDLN, and TIL of cancer patients

It has been described that the expression of CD25 and/or CD127 can be altered in (chronic) inflammatory/autoimmune diseases such as systemic lupus erythematosus (SLE) and type 1 diabetes, thereby influencing reliable Treg enumeration [12, 26, 31]. In addition, changes in CD25 and CD127 expressions have also been observed in cancer patients undergoing immunotherapeutic interventions such as vaccination or ipilimumab treatment [32–35]. To study the possibility of analyzing Tregs by the different definitions under such conditions, we analyzed peripheral blood samples from patients with recurrent OvCa and TDLN and tumor samples from CxCa patients.

**Fig. 3:**
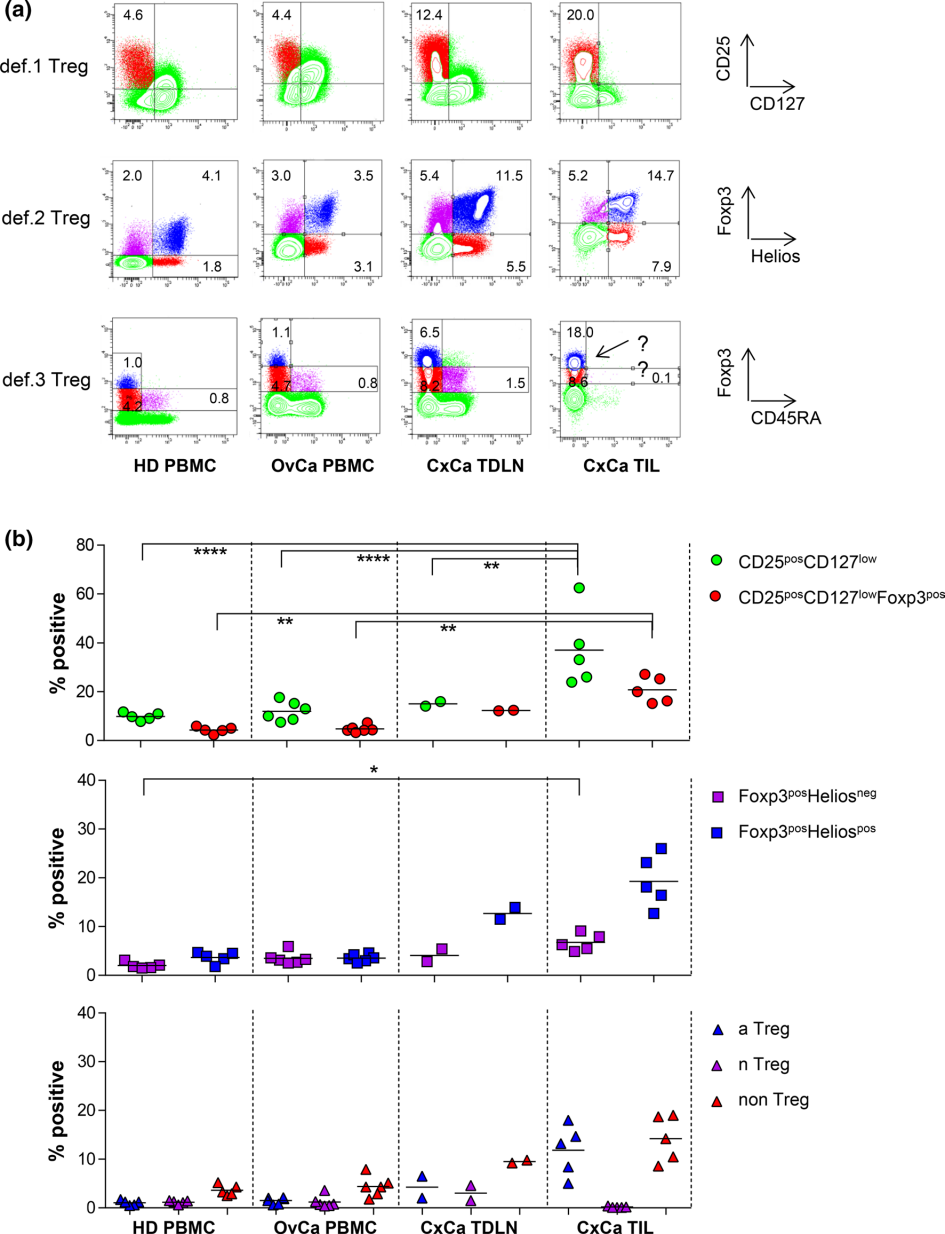
Treg gating based on Foxp3 and CD45RA (def.3) is subjective in TIL as it is difficult to distinguish between Foxp3^hi^ versus FoxP3^low^ cells due to the absence of Foxp3^int^CD45RA^pos^ population. Def.1, def.2, and def.3 Treg analyses were performed by flow cytometry. Treg analysis based on CD25 and CD127 (def.1), FoxP3 and Helios (def.2), and FoxP3 and CD45RA (def.3) is given for PBMC of a representative healthy donor (HD) and an ovarian cancer (OvCA) patient and for a TDLN and TIL sample of representative cervical cancer (CxCa) patient in **a** and for multiple donors in **b.** Gates were set as described in supplementary figures 2a, 3a, and 5a. Percentage of CD25^pos^CD127^low^ and CD25^pos^CD127^low^Foxp3^pos^ def.1; Foxp3^pos^Helios^neg^ and Foxp3^pos^Helios^pos^ def.2; and the def.3 Foxp3^hi^CD45RA^neg^ aTreg, Foxp3^int^CD45RA^pos^ nTreg, and Foxp3^int^CD45RA^neg^ non-Treg populations is given as percentage of CD3^pos^CD4^pos^ T cells. Example of the problem with gating based on Foxp3 and CD45RA in TIL is depicted by the *arrow* in **a**

As shown in Fig. 3 for representative examples, the gating and enumeration of Tregs based on CD25, CD127, Foxp3 (def.1), and Foxp3 and Helios (def.2) is feasible in OvCa-derived peripheral blood, as well as in TDLN and tumor samples from CxCa patients using the same gating strategy applied for HD-derived PBMC. Treg enumeration based on def.3 was feasible in peripheral blood and TDLN samples of patients but was not reliable in tumor samples due to the absence of the Foxp3^int^CD45RA^pos^ nTreg population which is used for discrimination between Foxp3^int^ and Foxp3^hi^ cells in the gating strategy (see also Supplementary figure 5 for Foxp3 and CD45RA gating strategy). Figure 3b shows a summary of the detected Treg frequencies in all analyzed samples. Importantly, the overlap between the three Treg subsets was comparable between HD-derived and OvCa patient-derived peripheral blood, CxCa-derived TDLN and tumor samples (Supplementary figure 9), indicating that CD25, CD127, and Foxp3 can also be used in cancer condition tissues. Of note, the additional value of CD127 and CD25 in the def.1 Treg marker set becomes particularly clear upon exclusion of these markers when assessing Treg frequencies in these samples. Exclusion of CD127 and/or CD25 from the Treg panel resulted in increase in the number of detected def.1 Tregs (supplementary figure 10). Although exclusion of CD127 only led to a substantial increase in the frequency of def.1 Tregs (mean 21.5 %, range 14.7–29.3 %) in the PBMC of OvCa patients, exclusion of CD25 or CD25 and CD127 led to substantial increases in the frequency of these def.1 Tregs in PBMC of HD and OvCa patients as well as in TDLN or tumor samples from CxCa patients (17.9, 21.9, 24.0, and 30.8 % for CD25 exclusion and 37.6, 58.8, 40.4, and 43.3 % for CD25 and CD127 exclusions, see supplementary figure 10a). This resulted from a less pure Treg detection as reflected by lower percentage of def.1 Tregs expressing markers such as CTLA-4 and reduced frequencies of Helios^pos^ def.2 and Foxp3^hi^CD45RA^neg^ def.3 aTreg cells among the def.1 Tregs (supplementary figure 10b), indicating that CD25 and CD127 are required for reliable assessment of def.1 Tregs.

### The association between Tregs and survival

**Fig. 4:**
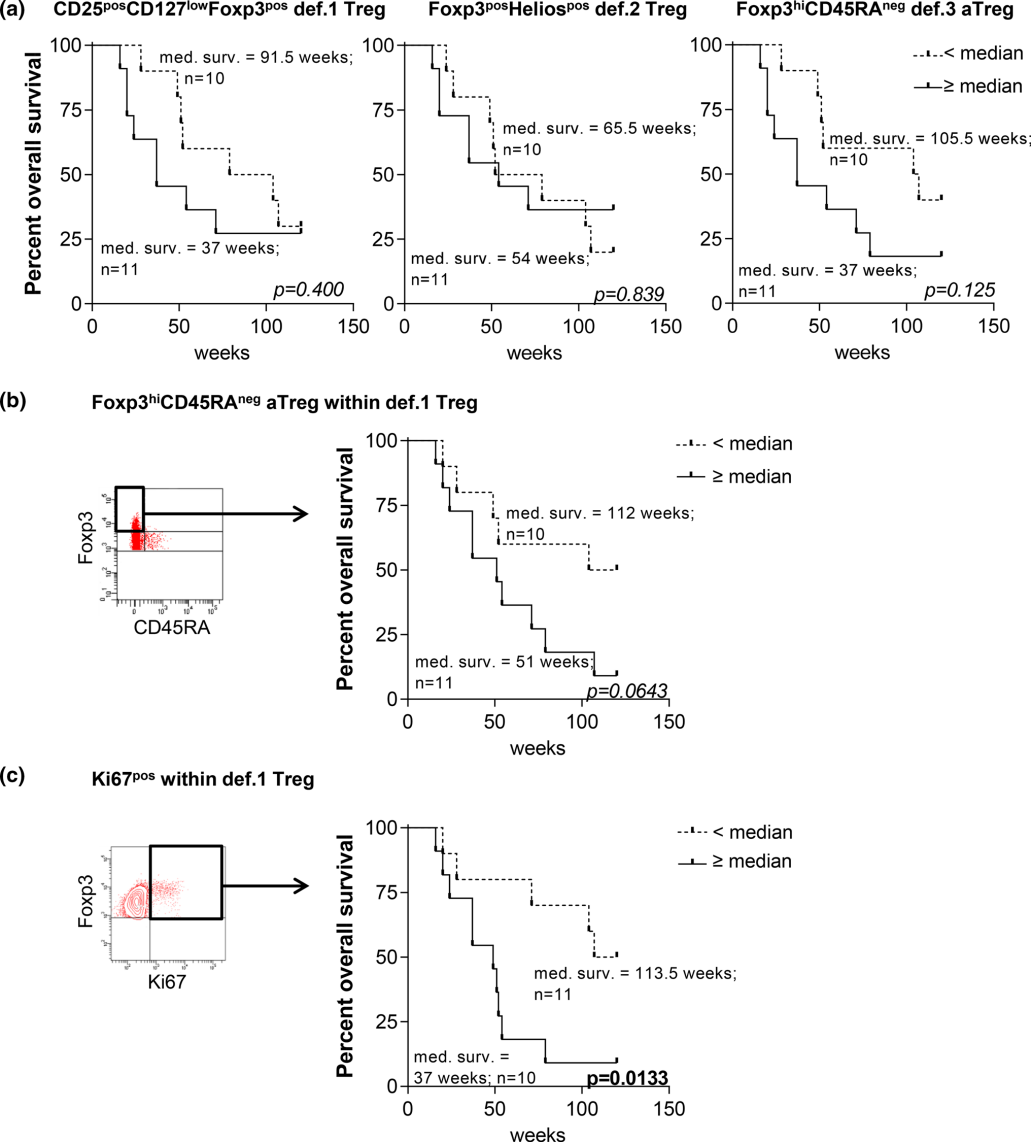
High pretreatment frequencies of Foxp3^hi^CD45RA^neg^ and Ki67^pos^ def.1 Tregs (i.e., activated def.1 Tregs) are associated with reduced overall survival in OvCa patients undergoing chemo-immunotherapeutic therapy. The use of Ki67 and CD45RA provides additional information on the activation status of def.1 Tregs. Treg analysis was performed based on CD25, CD127, and Foxp3 (def.1), Foxp3 and Helios (def.2), and Foxp3 and CD45RA (def.3) in PBMC of 21 chemo-immunotherapy-treated ovarian cancer (OvCA) patients (EM Dijkgraaf et al., submitted for publication). Pretreatment values of def.1, def.2, and def.3 Tregs were determined, and overall survival (OS) of these patients following chemo-immunotherapy was plotted in Kaplan–Meier curves for pretreatment values of def.1 (*left*), def.2 (*middle*), and def.3 (*right*) Tregs in **a**. Activation status of def.1 Tregs was determined by measuring the frequency of Foxp3^hi^CD45RA^neg^ and Ki67^pos^ cells within the def.1 Tregs. Gating and Kaplan–Meier curves are depicted in **b** for pretreatment values of Foxp3^hi^CD45RA^neg^ def.1 Tregs and **c** for pretreatment values of Ki67^pos^ def.1 Tregs. Gates for Foxp3^hi^CD45RA^neg^ and Ki67^pos^ were set as shown in the FACS plots. Patients were grouped into two groups based on the median of the total population, i.e., into a group of patients with frequencies that were below the median (*dotted line*) or with frequencies above the median (*solid line*) for the indicated parameter, after which survival analysis was performed. Number of patients and corresponding OS for each group is given. Statistical analysis was performed by log-rank testing, and differences were considered significant when *p* < 0.05

Treg accumulation in the tumor or peripheral blood is associated with tumor progression and poor prognosis [3–6]. To study the relation between the different Treg subsets and survival, we determined the frequencies of the def.1, def.2, and def.3 Tregs in the PBMC of recurrent OvCa patients undergoing chemo-immunotherapeutic treatment (EM Dijkgraaf et al. submitted for publication) and correlated these levels to the overall survival (OS). Pretreatment levels of none of the def.1 Tregs, def.2 Tregs, and def.3 aTreg correlated with survival (Fig. 4a). However, when the pretreatment frequencies of Foxp3^hi^CD45RA^neg^ or Ki67^pos^ cells within def.1 Tregs (i.e., activated def.1 Tregs) were determined, a trend toward reduced OS was observed for patients with high frequencies of Foxp3^hi^CD45RA^neg^ def.1 Tregs (*p* = 0.0643) and a significant reduced OS for patients with high frequencies of Ki67^pos^ def.1 Tregs (*p* = 0.0133; Fig. 4b). The latter suggests that in particular, measurements of a more activated Treg pool may have prognostic or predictive value.

## Conclusion and discussion

The unambiguous enumeration of Tregs by flow cytometry is hampered by (a) the inability to directly measure their function and (b) the absence of an exclusive, highly specific marker. Reaching consensus on an essential marker set for Treg enumeration with the currently available markers involves a number of considerations. First, the essential marker set should be able to identify a population of cells that in addition to the essential Treg-defining markers also express other Treg-associated markers but do not produce IFNγ and IL-2 [12, 13, 26]. Secondly, as there are currently three Treg definitions used in the field [8–12, 26], the cell population identified should be highly specific and include at least the same population of Tregs by all three definitions. Third, the proposed marker set should allow for robust, undisputable, and context (tissue)-independent gating since differences in gating strategies have been found to be the biggest source for interassay variation in flow cytometry-based assays [16, 17]. Fourth, if possible, one should be able to assess their functionality.

Based on the data presented here and taking into account the above-mentioned considerations, we consider the use of the CD3, CD4, CD25, CD127, and Foxp3 markers as the minimally required markers to define human Tregs. We showed that this combination of markers allows for robust and undisputable gating of Tregs in the context of HD- and cancer patient-derived peripheral blood as well as TDLN and tumor samples (Supplementary figure 2 and Fig. 3). Although the latter also holds true for Foxp3^pos^Helios^pos^ def.2 Tregs (Supplementary figure 3 and Fig. 3), Treg measurement based solely on Foxp3 and Helios resulted in a ~25 % underestimation of the number of def.1 Tregs through exclusion of CD25^pos^CD127^low^ cells within the Foxp3^pos^Helios^neg^ population in all tested tissues/compartments (supplementary figure 4 and 9). These observations were in line with findings from others, reporting that Helios expression was restricted to a subpopulation (approximately 70 %) of human Foxp3^pos^ T(reg) cells [12, 13, 26]. Treg enumeration based on Foxp3 and CD45RA (def.3) yielded distinctive aTreg and nTreg populations in HD- and cancer patient-derived peripheral blood and TDLN, with high CD25, CTLA-4, and Ki67 expression levels in the aTreg and lower expression levels of these markers in the nTreg populations (Supplementary figure 6 and figure 1 and 3). Yet, in line with findings from others [12], the largest population of CD25^pos^CD127^low^Foxp3^pos^ (def.1; supplementary figure 6c) or Foxp3^pos^Helios^pos^ (def.2; supplementary figure 6e) populations was found in the so-called non-Treg population of Foxp3^int^CD45RA^neg^ cells. While the population of Tregs based on definitions 1 or 2 may contain small fractions on non-Tregs, the measurement of Tregs based solely on Foxp3 and CD45RA (def.3) will lead to a ~60–70 % underestimation of Tregs. Importantly, def.3 Treg gating could not be done in a robust and undisputable fashion in tumor samples. Although not unexpected and observed before [8], the absence of the Foxp3^int^CD45RA^pos^ T cell population in tumor samples precluded robust def.3 aTreg and nTreg gatings in this context. Notably, the apparent absence of naïve T cells at tumor effector sites and the preferential recruitment of activated Tregs or accumulation of locally activated Tregs does confirm the validity of the defined respective activated and naïve Treg definitions within definition 3 [8, 11]. Of note, this observation clearly emphasizes the need for validating/assessing the suitability of the flow cytometry panels in the intended context/tissue.

As shown, we used CD3^pos^CD4^neg^ (i.e., CD8^pos^) and CD3^neg^ cells to define the limits of the positive (CD25, CD127, Helios, and CD45RA) gates as this has been described to form a more reliable gating strategy than using isotype control antibodies or FMO controls [23]. Omission of CD3 and CD8 antibodies from the essential marker set does affect our gating strategy resulting in less reliable/more disputable CD25, CD127, Helios, and CD45RA gating, and thus affecting the reliability of our results (data not shown). Furthermore, this gating strategy results in objective CD25^pos^ gating rather than subjective CD25^high^ gating, the latter being very important for harmonized and comparative Treg analysis.

There are a number of Treg-associated markers which we consider to be of interest, yet optional to the required minimal panel. Based on our data, we highly recommend extending the minimally required antibody panel to include Ki67 and CD45RA as they provide additional information on the Treg activation status (Table 2). Indeed, the addition of CD45RA and Ki67 to the marker panel proved very informative in that no def.1, def.2, or def.3 Tregs were associated with worse survival of ovarian cancer patients but only the pretreatment frequencies of activated Foxp3^pos^CD45RA^neg^ and Ki67^pos^ def.1 Tregs (Fig. 4). The measurement of activated Ki67^pos^ Tregs has also been advocated by others [36, 37]. In one study, renal cell cancer patients undergoing multipeptide vaccination and cyclophosphamide treatment showed a significant reduction in the number of circulation Ki67^pos^ Tregs and a trend toward prolonged OS following therapy [37]. Of note, as Ki67^pos^ def.1 Treg detection was also feasible in TDLN and tumor samples (not shown), this strategy may also be useful to identify activated Tregs within def.1 Tregs in tumor samples, thereby circumventing the need for the subjective gating on Foxp3^hi^ versus Foxp3^int^ cells. While the activation markers CD39 and CTLA-4 [27, 28, 38, 39] have been described as functional markers to identify activated Tregs, they do not provide additional information to a panel over CD45RA and Ki67 and the minimally required antibody set. Helios may be of interest for identifying Tregs in autoimmunity such as SLE since these patients’ conventional T cells display high levels of CD25 resulting in overlap with Tregs [12]. In a recent trial where patients displayed a strong antigen-specific CD4^pos^ T cell response to vaccination, we did not observe such problems for identifying Tregs using the currently proposed markers (EM Dijkgraaf et al. submitted for publication). Based on our data, omission of CD25 as a marker is not recommended as this resulted in the identification of less pure Treg populations (Supplementary figure 10).

**Table 2:** Proposed marker set

Order^a^	Marker	Expression	Location	Advice	Comment
1	CD3	Directly ex vivo	Cell surface	Essential	
2	CD4	Directly ex vivo	Cell surface	Essential	
3	CD25	Directly ex vivo	Cell surface	Essential	
4	Foxp3	Directly ex vivo	Intranuclear	Essential	
5	CD127	Directly ex vivo; low/absent	Cell surface	Essential	
6	Ki67	Directly ex vivo	Intranuclear	Highly recommended	In recently activated/proliferating Tregs
7	CD45RA	Directly ex vivo	Cell surface	Highly recommended	Discriminates between naïve and TCR-triggered Tregs
8	CTLA4	Directly ex vivo	Intracellular	Optional	On (previously) activated Tregs
9	Helios	Directly ex vivo	Intranuclear	Optional	Superior to CD25/CD127 in autoimmune conditions (such as SLE)
10	CD39	Directly ex vivo	Cell surface	Optional	Present on suppressive Tregs
11	LAP/GARP	Upon activation (>24 h) on PBMC/directly ex vivo on TIL	Intracellular	Optional	On activated Tregs

^a^Proposed order based on the outcome of the CIP workshop on the detection and functional testing of (antigen specific) regulatory T cells, subsequent discussions with leading experts in the field and data presented here

In addition, there remains a number of markers, not tested in this study, which may offer benefits to identify specific subsets of Tregs. CD147 is a cell surface marker that is accessible directly ex vivo and can also be used to identify an activated and highly suppressive Treg subset [36, 40, 41]. Furthermore, LAP (membrane-bound active form of TGF-β) and GARP (membrane-anchoring molecule involved in latent TGF-β binding) may be particularly interesting in defining TGF-β-associated and activated Tregs in tumor samples [39, 42–45]. Moreover, the chemokine receptors CCR6, CXCR3, CCR4, and CCR10 were found to be useful for the identification of phenotypical and functional distinct subsets of human Foxp3+ Tregs [46].

In summary, consensus was reached concerning the use of an essential marker set comprising antibodies to CD3, CD4, CD25, CD127, Foxp3, Ki67, and CD45RA and a corresponding robust gating strategy for the analysis of Tregs in human samples. This set will be used in proficiency panels to harmonize the phenotypic analysis of Tregs within laboratories participating in the CIP.

### Electronic supplementary material

Below is the link to the electronic supplementary material.

Supplementary material 1 (PDF 1144 kb)

## Appendix

1. J. Albrecht—Helmholtz Zentrum München, Munich, Germany
2. N. Anfossi—Platine Pharma Services, Lyon, France
3. I. Bigalke—Oslo University Hospital, Oslo, Norway
4. C. Bosch-Voskens—University of Erlangen, Erlangen, Germany
5. A. ten Brinke—Sanquin, Amsterdam, The Netherlands
6. L. Brix—Immudex, Copenhagen, Denmark
7. T. Duiveman-de Boer—Nijmegen Center for Molecular Life Sciences (NCMLS), Radboud University Medical Center, Nijmegen, The Netherlands
8. J. Eckl—Helmholtz Zentrum München, Munich, Germany. ^*^Current affiliation: Medigene Immunotherapies GmbH, Planegg/Martinsried, Germany
9. S. Gross—University of Erlangen, Erlangen, Germany
10. C.M. Huijts—Vrije Universiteit (VU) University medical center, Amsterdam, The Netherlands
11. M. Idorn—Center for Cancer Immune Therapy, Herlev, Denmark
12. E.M. Inderberg Suso—Oslo University Hospital, Oslo, Norway
13. H. Jacobs—Radboud University Medical Centre, Nijmegen, The Netherlands
14. K. Laske—University of Tübingen, Tübingen, Germany
15. F.S. Lichtenegger—Klinikum der Universität München, Munich, Germany
16. D. Maurer—Immatics Biotechnologies GmbH, Tübingen, Germany
17. T. van Oorschot—Nijmegen Center for Molecular Life Sciences (NCMLS), Radboud University Medical Center, Nijmegen, The Netherlands
18. D. Schendel—Helmholtz Zentrum München, Munich, Germany. ^*^Current affiliation: Medigene Immunotherapies GmbH, Planegg/Martinsried, Germany
19. P.T. Straten—Center for Cancer Immune Therapy, Herlev, Denmark
20. F. Toulza—London School of Hygiene &Tropical Medicine, London, UK
21. A. Visser—Sanquin, Amsterdam, The Netherlands

## Abbreviations

ATP: Adenosine triphosphate
aTreg: Activated Treg
CIP: CIMT immunoguiding Program
CxCa: Cervical cancer
FMO: Fluorescence minus one
HD: Healthy donors
ICS: Intracellular cytokine staining
LN: Lymph node
MIATA: Minimal information about T cell assays
nTreg: Naïve Tregs
OS: Overall survival
OvCa: Ovarian cancer
SLE: Systemic lupus erythematosus
SOP: Standard operation procedures
TDLN: Tumor-draining lymph nodes
TIL: Tumor-infiltrating lymphocyte
Treg: Regulatory T cell

## References

[1] Grant CR, Liberal R, Mieli-Vergani G, Vergani D, Longhi MS (2014). Regulatory T-cells in autoimmune diseases: challenges, controversies and-yet-unanswered questions. Autoimmun Rev.

[2] Smigiel KS, Srivastava S, Stolley JM, Campbell DJ (2014). Regulatory T-cell homeostasis: steady-state maintenance and modulation during inflammation. Immunol Rev.

[3] Whiteside TL (2014). Regulatory T cell subsets in human cancer: are they regulating for or against tumor progression?. Cancer Immunol Immunother.

[4] Piersma SJ, Welters MJ, van der Burg SH (2008). Tumor-specific regulatory T cells in cancer patients. Hum Immunol.

[5] Welters MJ, Piersma SJ, van der Burg SH (2008). T-regulatory cells in tumour-specific vaccination strategies. Expert Opin Biol Ther.

[6] Petrausch U, Poehlein CH, Jensen SM, Twitty C, Thompson JA, Assmann I, Puri S, LaCelle MG, Moudgil T, Maston L, Friedman K, Church S, Cardenas E, Haley DP, Walker EB, Akporiaye E, Weinberg AD, Rosenheim S, Crocenzi TS, Hu HM, Curti BD, Urba WJ, Fox BA (2009). Cancer immunotherapy: the role regulatory T cells play and what can be done to overcome their inhibitory effects. Curr Mol Med.

[7] Whiteside TL (2012). What are regulatory T cells (Treg) regulating in cancer and why?. Semin Cancer Biol.

[8] Sugiyama D, Nishikawa H, Maeda Y, Nishioka M, Tanemura A, Katayama I, Ezoe S, Kanakura Y, Sato E, Fukumori Y, Karbach J, Jager E, Sakaguchi S (2013). Anti-CCR4 mAb selectively depletes effector-type FoxP3+ CD4+ regulatory T cells, evoking antitumor immune responses in humans. Proc Natl Acad Sci USA.

[9] Liu W, Putnam AL, Xu-Yu Z, Szot GL, Lee MR, Zhu S, Gottlieb PA, Kapranov P, Gingeras TR, Fazekas de St GB, Clayberger C, Soper DM, Ziegler SF, Bluestone JA (2006). CD127 expression inversely correlates with FoxP3 and suppressive function of human CD4+ T reg cells. J Exp Med.

[10] Seddiki N, Santner-Nanan B, Martinson J, Zaunders J, Sasson S, Landay A, Solomon M, Selby W, Alexander SI, Nanan R, Kelleher A, Fazekas de St GB (2006). Expression of interleukin (IL)-2 and IL-7 receptors discriminates between human regulatory and activated T cells. J Exp Med.

[11] Miyara M, Yoshioka Y, Kitoh A, Shima T, Wing K, Niwa A, Parizot C, Taflin C, Heike T, Valeyre D, Mathian A, Nakahata T, Yamaguchi T, Nomura T, Ono M, Amoura Z, Gorochov G, Sakaguchi S (2009). Functional delineation and differentiation dynamics of human CD4+ T cells expressing the FoxP3 transcription factor. Immunity.

[12] Golding A, Hasni S, Illei G, Shevach EM (2013). The percentage of FoxP3+ Helios+ Treg cells correlates positively with disease activity in systemic lupus erythematosus. Arthritis Rheum.

[13] Thornton AM, Korty PE, Tran DQ, Wohlfert EA, Murray PE, Belkaid Y, Shevach EM (2010). Expression of Helios, an Ikaros transcription factor family member, differentiates thymic-derived from peripherally induced Foxp3+ T regulatory cells. J Immunol.

[14] Mandapathil M, Hilldorfer B, Szczepanski MJ, Czystowska M, Szajnik M, Ren J, Lang S, Jackson EK, Gorelik E, Whiteside TL (2010). Generation and accumulation of immunosuppressive adenosine by human CD4+ CD25highFOXP3+ regulatory T cells. J Biol Chem.

[15] Loza MJ, Anderson AS, O’Rourke KS, Wood J, Khan IU (2011). T-cell specific defect in expression of the NTPDase CD39 as a biomarker for lupus. Cell Immunol.

[16] McNeil LK, Price L, Britten CM, Jaimes M, Maecker H, Odunsi K, Matsuzaki J, Staats JS, Thorpe J, Yuan J, Janetzki S (2013). A harmonized approach to intracellular cytokine staining gating: results from an international multiconsortia proficiency panel conducted by the cancer immunotherapy consortium (CIC/CRI). Cytometry A.

[17] Welters MJ, Gouttefangeas C, Ramwadhdoebe TH, Letsch A, Ottensmeier CH, Britten CM, van der Burg SH (2012). Harmonization of the intracellular cytokine staining assay. Cancer Immunol Immunother.

[18] Britten CM, Janetzki S, Butterfield LH, Ferrari G, Gouttefangeas C, Huber C, Kalos M, Levitsky HI, Maecker HT, Melief CJ, O’Donnell-Tormey J, Odunsi K, Old LJ, Ottenhoff TH, Ottensmeier C, Pawelec G, Roederer M, Roep BO, Romero P, van der Burg SH, Walter S, Hoos A, Davis MM (2012). T cell assays and MIATA: the essential minimum for maximum impact. Immunity.

[19] Singh SK, Meyering M, Ramwadhdoebe TH, Stynenbosch LF, Redeker A, Kuppen PJ, Melief CJ, Welters MJ, van der Burg SH (2012). The simultaneous ex vivo detection of low-frequency antigen-specific CD4+ and CD8+ T-cell responses using overlapping peptide pools. Cancer Immunol Immunother.

[20] Heusinkveld M, Welters MJ, van Poelgeest MI, van der Hulst JM, Melief CJ, Fleuren GJ, Kenter GG, van der Burg SH (2011). The detection of circulating human papillomavirus-specific T cells is associated with improved survival of patients with deeply infiltrating tumors. Int J Cancer.

[21] de Vos van Steenwijk PJ, Heusinkveld M, Ramwadhdoebe TH, Lowik MJ, van der Hulst JM, Goedemans R, Piersma SJ, Kenter GG, van der Burg SH (2010). An unexpectedly large polyclonal repertoire of HPV-specific T cells is poised for action in patients with cervical cancer. Cancer Res.

[22] Law JP, Hirschkorn DF, Owen RE, Biswas HH, Norris PJ, Lanteri MC (2009). The importance of Foxp3 antibody and fixation/permeabilization buffer combinations in identifying CD4+ CD25+ Foxp3+ regulatory T cells. Cytometry A.

[23] Presicce P, Moreno-Fernandez ME, Lages CS, Orsborn KI, Chougnet CA (2010). Association of two clones allows for optimal detection of human FOXP3. Cytometry A.

[24] Grant J, Bourcier K, Wallace S, Pan D, Conway A, Seyfert-Margolis V, Wallace PK (2009). Validated protocol for FoxP3 reveals increased expression in type 1 diabetes patients. Cytometry B Clin Cytom.

[25] Murdoch DM, Staats JS, Weinhold KJ (2012). OMIP-006: phenotypic subset analysis of human T regulatory cells via polychromatic flow cytometry. Cytometry A.

[26] Alexander T, Sattler A, Templin L, Kohler S, Gross C, Meisel A, Sawitzki B, Burmester GR, Arnold R, Radbruch A, Thiel A, Hiepe F (2013). Foxp3+ Helios+ regulatory T cells are expanded in active systemic lupus erythematosus. Ann Rheum Dis.

[27] Deaglio S, Dwyer KM, Gao W, Friedman D, Usheva A, Erat A, Chen JF, Enjyoji K, Linden J, Oukka M, Kuchroo VK, Strom TB, Robson SC (2007). Adenosine generation catalyzed by CD39 and CD73 expressed on regulatory T cells mediates immune suppression. J Exp Med.

[28] Schuler PJ, Schilling B, Harasymczuk M, Hoffmann TK, Johnson J, Lang S, Whiteside TL (2012). Phenotypic and functional characteristics of CD4+ CD39+ FOXP3+ and CD4+ CD39+ FOXP3neg T-cell subsets in cancer patients. Eur J Immunol.

[29] Mandapathil M, Lang S, Gorelik E, Whiteside TL (2009). Isolation of functional human regulatory T cells (Treg) from the peripheral blood based on the CD39 expression. J Immunol Methods.

[30] Schuler PJ, Harasymczuk M, Schilling B, Lang S, Whiteside TL (2011). Separation of human CD4+ CD39+ T cells by magnetic beads reveals two phenotypically and functionally different subsets. J Immunol Methods.

[31] Moniuszko M, Glowinska-Olszewska B, Rusak M, Jeznach M, Grubczak K, Lipinska D, Milewski R, Milewska AJ, Dabrowska M, Jablonska E, Kretowski A, Gorska M, Bodzenta-Lukaszyk A, Bossowski A (2013). Decreased CD127 expression on CD4+ T-cells and elevated frequencies of CD4+ CD25+ C. Clin Dev Immunol.

[32] Welters MJ, Kenter GG, Piersma SJ, Vloon AP, Lowik MJ, Berends-van der Meer DM, Drijfhout JW, Valentijn AR, Wafelman AR, Oostendorp J, Fleuren GJ, Offringa R, Melief CJ, van der Burg SH (2008). Induction of tumor-specific CD4+ and CD8+ T-cell immunity in cervical cancer patients by a human papillomavirus type 16 E6 and E7 long peptides vaccine. Clin Cancer Res.

[33] Welters MJ, Kenter GG, de Vos van Steenwijk PJ, Lowik MJ, Berends-van der Meer DM, Essahsah F, Stynenbosch LF, Vloon AP, Ramwadhdoebe TH, Piersma SJ, van der Hulst JM, Valentijn AR, Fathers LM, Drijfhout JW, Franken KL, Oostendorp J, Fleuren GJ, Melief CJ, van der Burg SH (2010). Success or failure of vaccination for HPV16-positive vulvar lesions correlates with kinetics and phenotype of induced T-cell responses. . Proc Natl Acad Sci USA.

[34] Kavanagh B, O’Brien S, Lee D, Hou Y, Weinberg V, Rini B, Allison JP, Small EJ, Fong L (2008). CTLA4 blockade expands FoxP3+ regulatory and activated effector CD4+ T cells in a dose-dependent fashion. Blood.

[35] Sun J, Tang DN, Fu T, Sharma P (2012). Identification of human regulatory T cells in the setting of T-cell activation and anti-CTLA-4 immunotherapy on the basis of expression of latency-associated peptide. Cancer Discov.

[36] Landskron J, Helland O, Torgersen KM, Aandahl EM, Gjertsen BT, Bjorge L, Tasken K (2014). Activated regulatory and memory T-cells accumulate in malignant ascites from ovarian carcinoma patients. Cancer Immunol Immunother.

[37] Walter S, Weinschenk T, Stenzl A, Zdrojowy R, Pluzanska A, Szczylik C, Staehler M, Brugger W, Dietrich PY, Mendrzyk R, Hilf N, Schoor O, Fritsche J, Mahr A, Maurer D, Vass V, Trautwein C, Lewandrowski P, Flohr C, Pohla H, Stanczak JJ, Bronte V, Mandruzzato S, Biedermann T, Pawelec G, Derhovanessian E, Yamagishi H, Miki T, Hongo F, Takaha N, Hirakawa K, Tanaka H, Stevanovic S, Frisch J, Mayer-Mokler A, Kirner A, Rammensee HG, Reinhardt C, Singh-Jasuja H (2012). Multipeptide immune response to cancer vaccine IMA901 after single-dose cyclophosphamide associates with longer patient survival. Nat Med.

[38] Battaglia M, Roncarolo MG (2009). The fate of human Treg cells. Immunity.

[39] Jie HB, Gildener-Leapman N, Li J, Srivastava RM, Gibson SP, Whiteside TL, Ferris RL (2013). Intratumoral regulatory T cells upregulate immunosuppressive molecules in head and neck cancer patients. Br J Cancer.

[40] Landskron J, Tasken K (2013). CD147 in regulatory T cells. Cell Immunol.

[41] Solstad T, Bains SJ, Landskron J, Aandahl EM, Thiede B, Tasken K, Torgersen KM (2011). CD147 (Basigin/Emmprin) identifies FoxP3+ CD45RO+ CTLA4+ -activated human regulatory T cells. Blood.

[42] Tran DQ, Andersson J, Wang R, Ramsey H, Unutmaz D, Shevach EM (2009). GARP (LRRC32) is essential for the surface expression of latent TGF-beta on platelets and activated FOXP3+ regulatory T cells. Proc Natl Acad Sci USA.

[43] Tran DQ, Andersson J, Hardwick D, Bebris L, Illei GG, Shevach EM (2009). Selective expression of latency-associated peptide (LAP) and IL-1 receptor type I/II (CD121a/CD121b) on activated human FOXP3+ regulatory T cells allows for their purification from expansion cultures. Blood.

[44] Scurr M, Ladell K, Besneux M, Christian A, Hockey T, Smart K, Bridgeman H, Hargest R, Phillips S, Davies M, Price D, Gallimore A, Godkin A (2014). Highly prevalent colorectal cancer-infiltrating LAP(+) Foxp3(−) T cells exhibit more potent immunosuppressive activity than Foxp3(+) regulatory T cells. Mucosal Immunol.

[45] Probst-Kepper M, Buer J (2010). FOXP3 and GARP (LRRC32): the master and its minion. Biol Direct.

[46] Duhen T, Duhen R, Lanzavecchia A, Sallusto F, Campbell DJ (2012). Functionally distinct subsets of human FOXP3+ Treg cells that phenotypically mirror effector Th cells. Blood.

[47] Gottschalk RA, Corse E, Allison JP (2012). Expression of Helios in peripherally induced Foxp3+ regulatory T cells. J Immunol.

[48] Himmel ME, MacDonald KG, Garcia RV, Steiner TS, Levings MK (2013). Helios+ and Helios− cells coexist within the natural FOXP3+ T regulatory cell subset in humans. J Immunol.

[49] Bacher P, Kniemeyer O, Schonbrunn A, Sawitzki B, Assenmacher M, Rietschel E, Steinbach A, Cornely OA, Brakhage AA, Thiel A, Scheffold A (2014). Antigen-specific expansion of human regulatory T cells as a major tolerance mechanism against mucosal fungi. Mucosal Immunol.

[50] Schoenbrunn A, Frentsch M, Kohler S, Keye J, Dooms H, Moewes B, Dong J, Loddenkemper C, Sieper J, Wu P, Romagnani C, Matzmohr N, Thiel A (2012). A converse 4-1BB and CD40 ligand expression pattern delineates activated regulatory T cells (Treg) and conventional T cells enabling direct isolation of alloantigen-reactive natural Foxp3+ Treg. J Immunol.

[51] Kleinewietfeld M, Starke M, Di MD, Borsellino G, Battistini L, Rotzschke O, Falk K (2009). CD49d provides access to “untouched” human Foxp3+ Treg free of contaminating effector cells. Blood.

[52] Battaglia A, Buzzonetti A, Baranello C, Ferrandina G, Martinelli E, Fanfani F, Scambia G, Fattorossi A (2009). Metastatic tumour cells favour the generation of a tolerogenic milieu in tumour draining lymph node in patients with early cervical cancer. Cancer Immunol Immunother.

[53] Delgoffe GM, Woo SR, Turnis ME, Gravano DM, Guy C, Overacre AE, Bettini ML, Vogel P, Finkelstein D, Bonnevier J, Workman CJ, Vignali DA (2013). Stability and function of regulatory T cells is maintained by a neuropilin-1-semaphorin-4a axis. Nature.

[54] Milpied P, Renand A, Bruneau J, Mendes-da-Cruz DA, Jacquelin S, Asnafi V, Rubio MT, Macintyre E, Lepelletier Y, Hermine O (2009). Neuropilin-1 is not a marker of human Foxp3+ Treg. Eur J Immunol.

