# Supplementary material for: Monitoring regulatory T cells in clinical samples: consensus on an essential marker set and gating strategy for regulatory T cell analysis by flow cytometry
Source: Cancer Immunol Immunother. 2015 Jun 28;64(10):1271–86. doi: 10.1007/s00262-015-1729-x (PMC4554737; doi:10.1007/s00262-015-1729-x)
Supplement: Supplementary file 1 — Supplementary material 1 (PDF 1144 kb) [file 262_2015_1729_MOESM1_ESM.pdf]

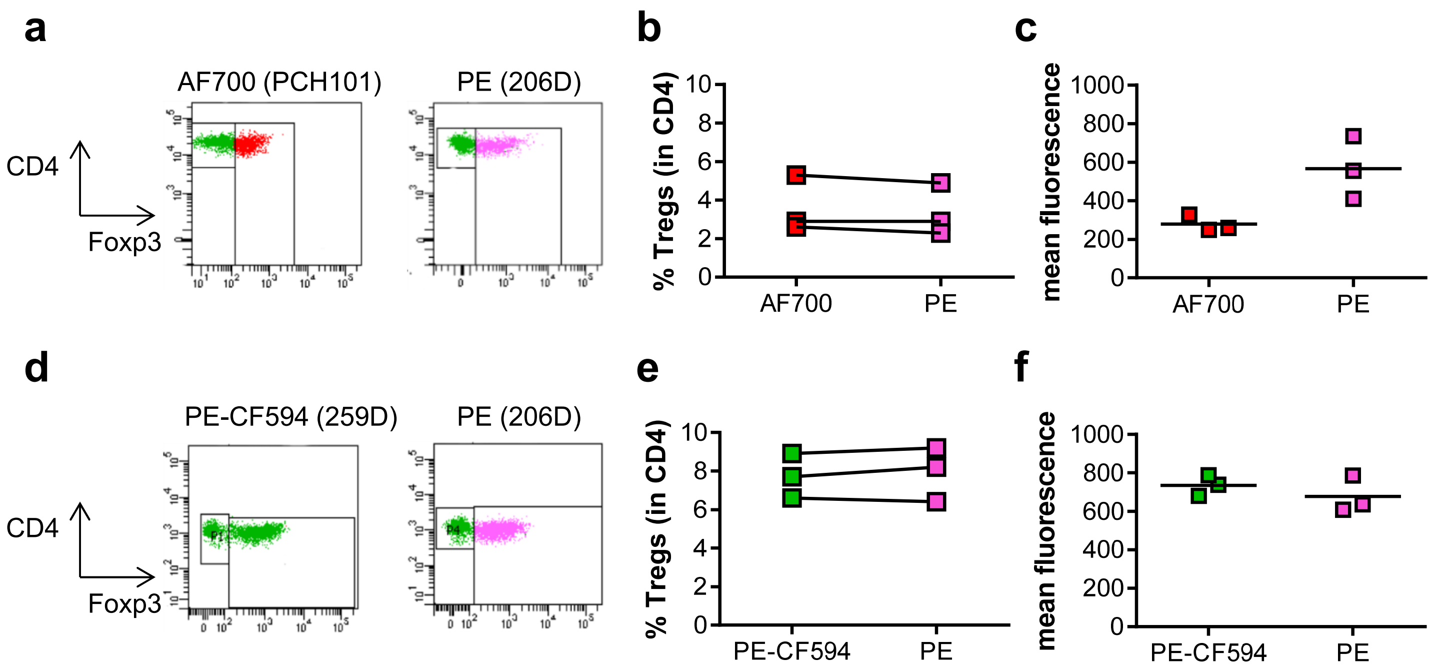

Supplementary figure 1. Panel set-up to determine optimal nuclear Foxp3 staining. **a)** PCH101-AF700 and 206D-PE Foxp3 staining was done with the eBiosciences Foxp3 staining buffer set, and is given for a representative example. Cells shown are gated for CD25^{pos}CD127^{low}, after which Foxp3 is depicted for the PCH101-AF700 (in red) and 206D-PE (in pink) antibody. Percentage Treg within CD4^{+} T cells **b)** and mean fluorescence of the Foxp3 signal of the CD25^{pos}CD127^{low} population **c)** is given for three donors with the PCH101-AF700 (red) and 206D-PE (pink) antibody. **d)** 206D-PE and 259D/C7-PE-CF594 Foxp3 staining was done with the BD TF buffer set, and is given for a representative example. Cells shown are gated for CD25^{pos}CD127^{low}, after which Foxp3 is depicted for the 259D/C7-PE-CF594 (in green) and 206D-PE (in pink) antibody. Percentage Treg within CD4^{+} T cells **e)** and mean fluorescence of the Foxp3 signal of the CD25^{pos}CD127^{low} population **f)** is given for three donors with the 259D/C7-PE-CF594 (green) and the 206D-PE (red) antibody.

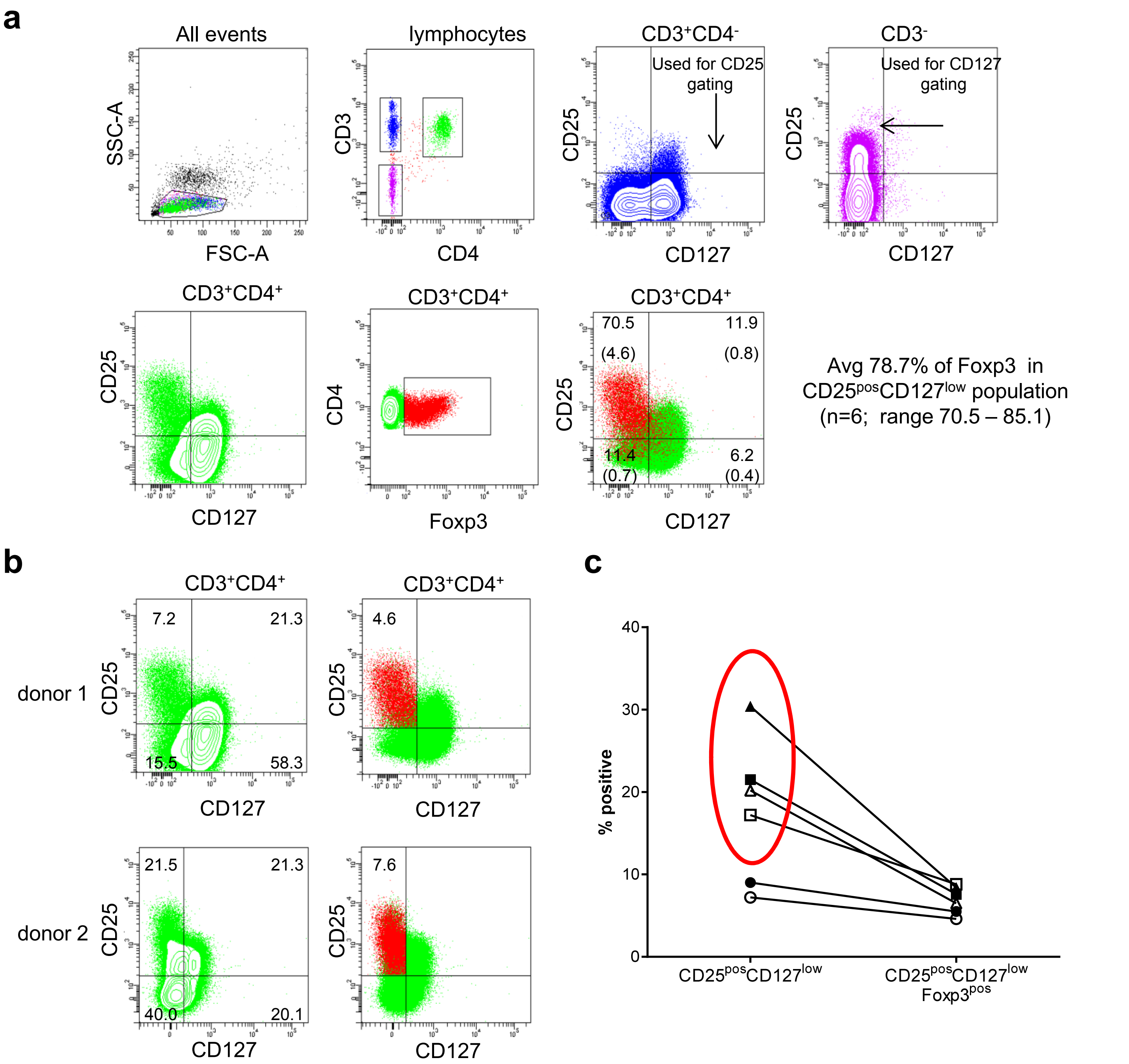

Supplementary figure 2. Treg analysis based on CD25, CD127 and Foxp3 (def.1). PBMC from 6 healthy donors (HD) were analyzed. **a)** Gating strategy for the CD25^{pos}CD127^{low}Foxp3^{pos} subset (def.1) is given for a representative HD. Gates for CD25 and CD127 positivity were set on CD3^{+}CD4^{-} (i.e. CD8+) T cells and CD3- lymphocytes respectively (top panel) and subsequently applied to CD3^{+}CD4^{+} T cells (lower panel). Foxp3 was gated on CD3^{+}CD4^{+} T cells, after which distribution of the Foxp3 was shown within the CD3^{+}CD4^{+} T cells. Distribution of Foxp3 is shown in red and given as percentage of cells within all the CD25CD127 populations and as percentage of CD4^{+} T cells for the CD25^{pos}CD127^{low} population in brackets. **b)** CD25CD127 gating for two different donors is depicted. Percentage of CD25^{pos}CD127^{low} and CD25^{pos}CD127^{low}Foxp3^{pos} cells within CD4^{+} T cells are given in the upper left quadrants. **c)** Percentage of CD25^{pos}CD127^{low} and CD25^{pos}CD127^{low}Foxp3^{pos} cells within CD3^{+}CD4^{+} T cells is depicted for six different HD.

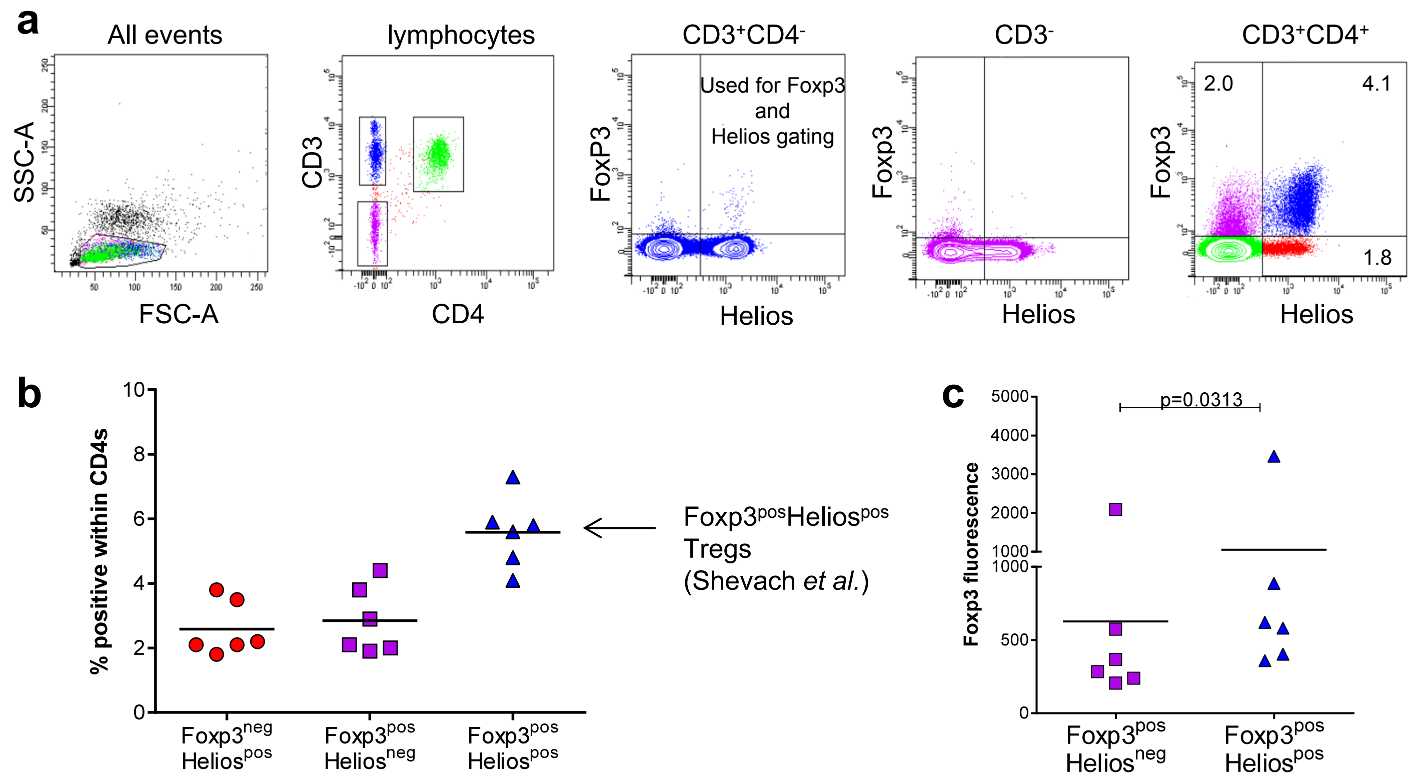

Supplementary figure 3. Treg analysis based on Foxp3 and Helios (def.2). PBMC from six healthy donors (HD) were analyzed. **a)** The gating strategy for the Foxp3^{pos}Helios^{pos} def.2 Treg subset is given for a representative HD. Gates for Foxp3 and Helios were set on CD3^{+}CD4^{-} (i.e. CD8^{+}) T cells and CD3^{-} lymphocytes respectively and applied to CD3^{+}CD4^{+} T cells. Percentage of Foxp3^{pos}Helios^{neg} , Foxp3^{pos}Helios^{pos} and Foxp3^{neg}Helios^{pos} in CD3^{+}CD4^{+} T cells is given for **a)** a representative HD and **b)** six HD. **c)** Foxp3 fluorescence is given for the Foxp3^{pos}Helios^{neg} and Foxp3^{pos}Helios^{pos} subsets for six HD.

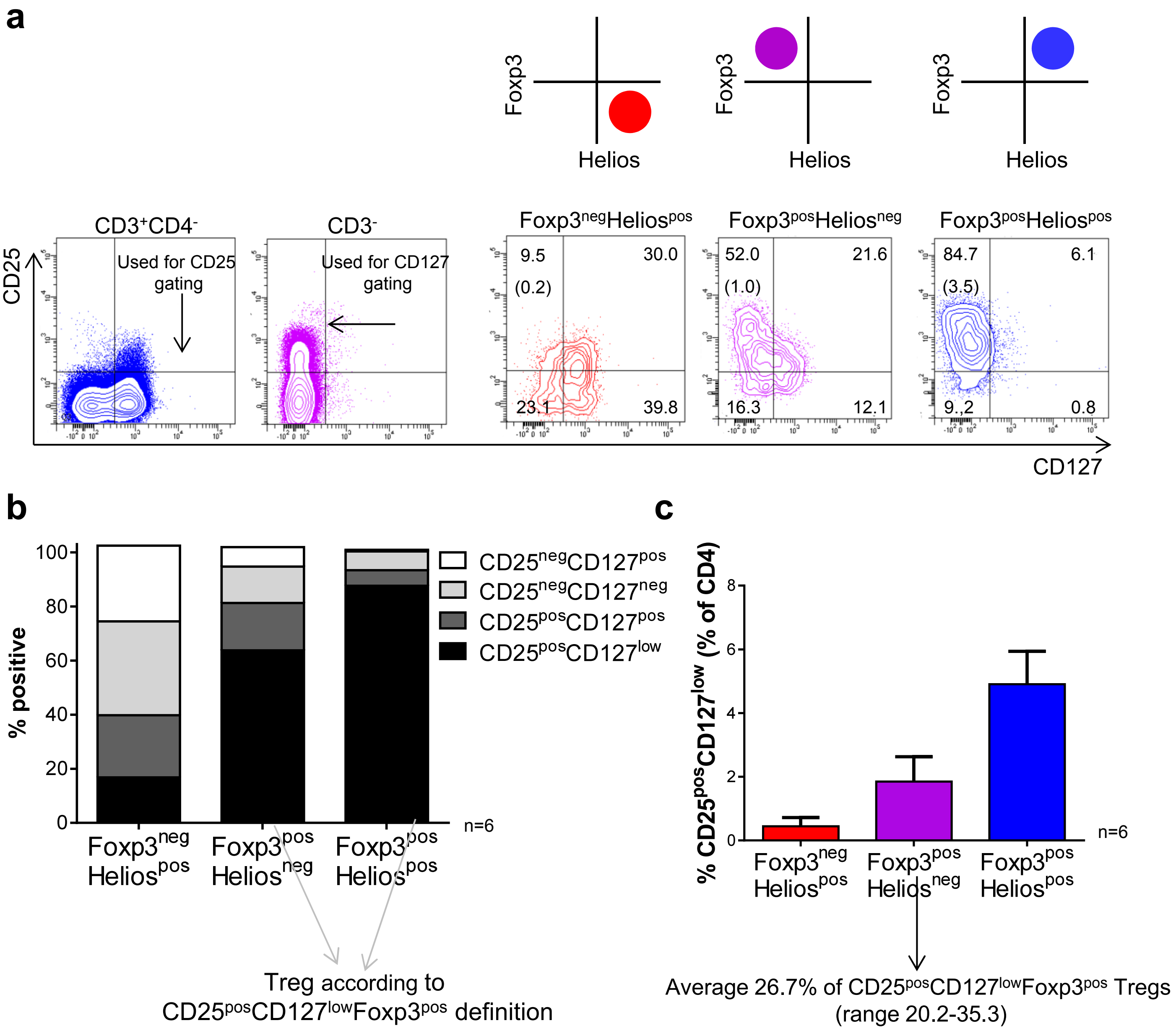

Supplementary figure 4. Phenotypic characterization of Foxp3posHeliosneg and Foxp3posHeliospos def.2 Treg subsets. PBMC from six healthy donors (HD) were analyzed. **a)** Gating strategy for the CD25posCD127low def.1 Treg subset within the Foxp3negHeliospos (red), Foxp3posHeliosneg (purple) and Foxp3posHeliospos (blue) populations is given. Gates for CD25 and CD127 were set on $CD3^+CD4^-$ (i.e. $CD8^+$) T cells and $CD3^-$ lymphocytes respectively (left panels) and applied to the three Foxp3Helios populations (right panels). Data shown are for a representative HD **(a)** and six HD **(b and c).** Percentage of CD25 and CD127 expression is given as percentage of cells within the given Foxp3Helios population **(a and b)** and as percentage of $CD4^+$ T cells for the indicated Foxp3Helios subset (**a** (in brackets) and **c**).

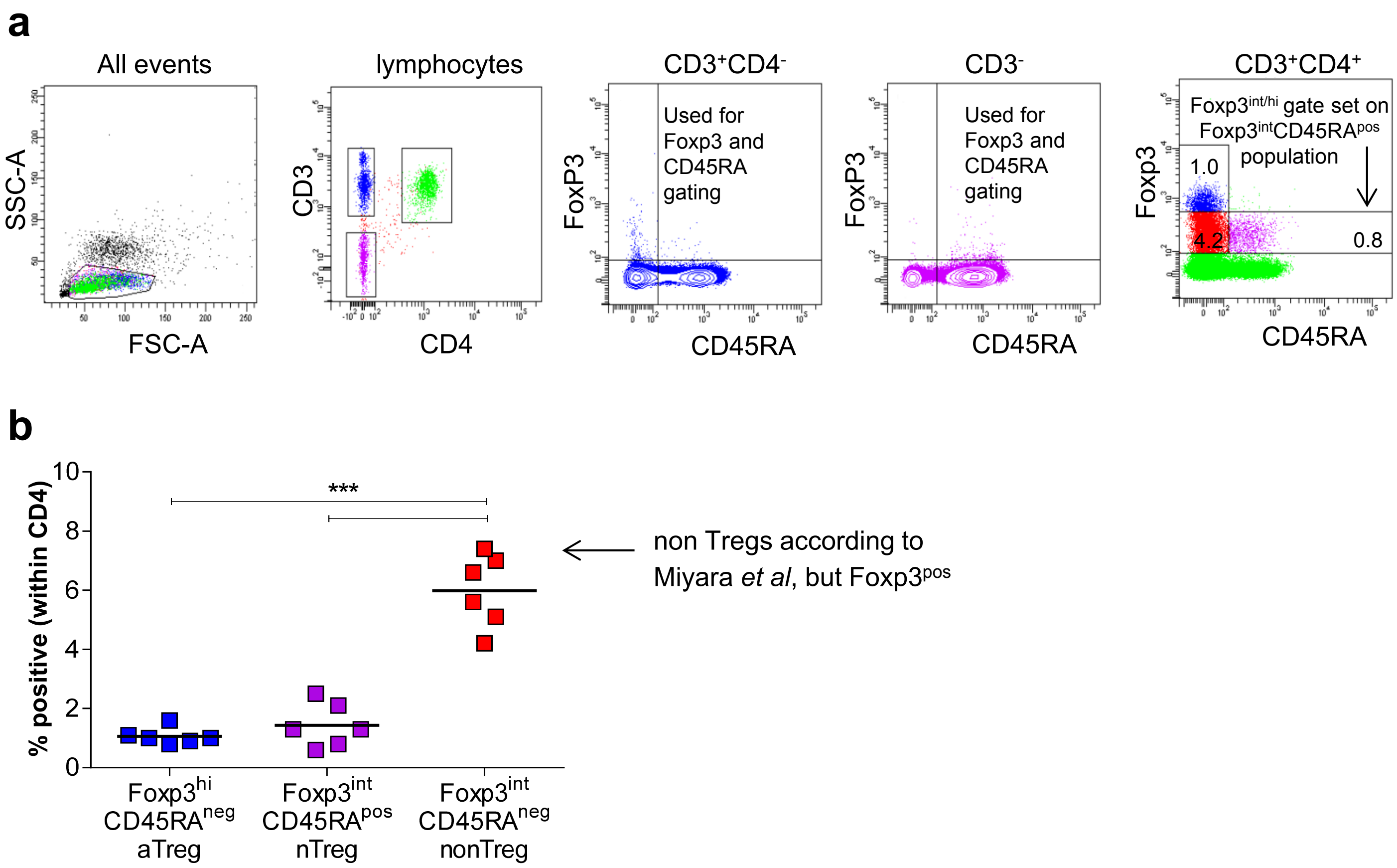

Supplementary figure 5. Treg analysis based on Foxp3 and CD45RA (def.3). PBMC from six healthy donors (HD) were analyzed. Data shown are for **a)** a representative HD and **b)** six HD. Gates for Foxp3$^{pos/neg}$ and CD45RA$^{pos/neg}$ were set on CD3^{+}CD4^{-} (i.e. CD8^{+}) T cells and CD3^{-} lymphocytes and copied to CD3^{+}CD4^{+} T cells. Gate for Foxp3$^{int/hi}$ was set on the Foxp3^{int}CD45RA^{pos} population. Percentage of Foxp3^{hi}CD45RA^{neg} activated (a) Treg (blue), Foxp3^{int}CD45RA^{pos} naïve (n) Treg (purple) and Foxp3^{int}CD45RA^{neg} non Treg (red) are given as percentage of CD4^{+} T cells.

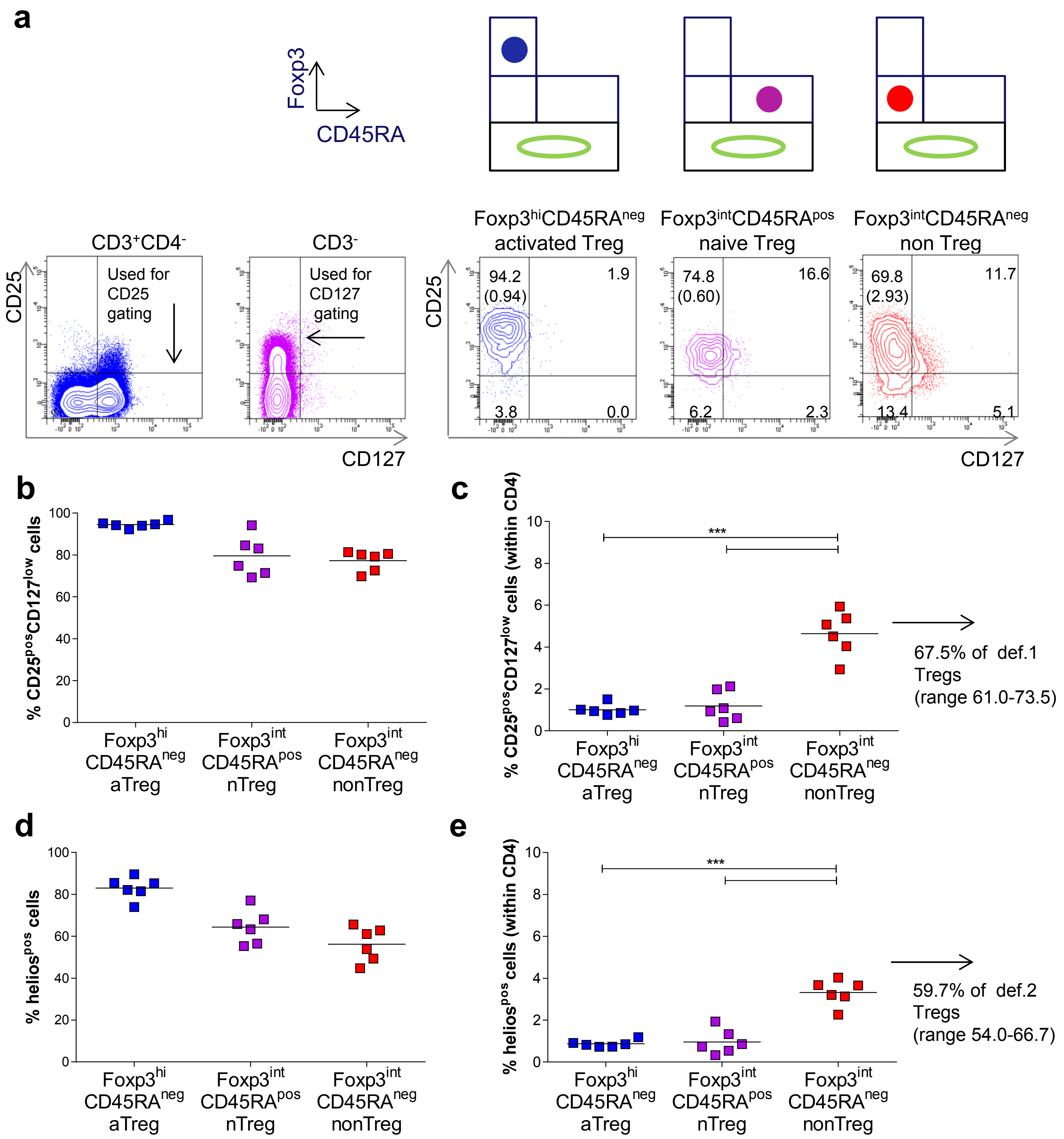

Supplementary figure 6. Phenotypic characterization of the Foxp3^{hi}CD45RA^{neg} aTreg, Foxp3^{int}CD45RA^{pos} nTreg and Foxp3^{int}CD45RA^{neg} non Treg subsets (def.3). a) Gating strategy for the CD25^{pos}CD127^{low} subset (def.1) within the Foxp3^{hi}CD45RA^{neg} aTreg (blue), Foxp3^{int}CD45RA^{pos} nTreg (purple) and Foxp3^{int}CD45RA^{neg} non Treg (red) populations is given. Gates for CD25 and CD127 were set on CD3^{+}CD4^{-} (i.e. CD8^{+}) T cells and CD3^{-} lymphocytes respectively (top panel) and applied to the three Foxp3CD45RA populations. Data shown are for a representative HD **(a)** and six HD **(b and c)**. Percentage of CD25^{pos}CD127^{low} cells is given as percentage of cells within the given Foxp3CD45RA populations **(a and b)** and as percentage of CD4^{+} T cells for the indicated Foxp3CD45RA subsets (**a** (in brackets) **and c**). Percentage of helios^{pos} cells is given as percentage of cells within the given Foxp3CD45RA populations **(d)** and as percentage of CD4^{+} T cells for the indicated Foxp3CD45RA subsets (**e**).

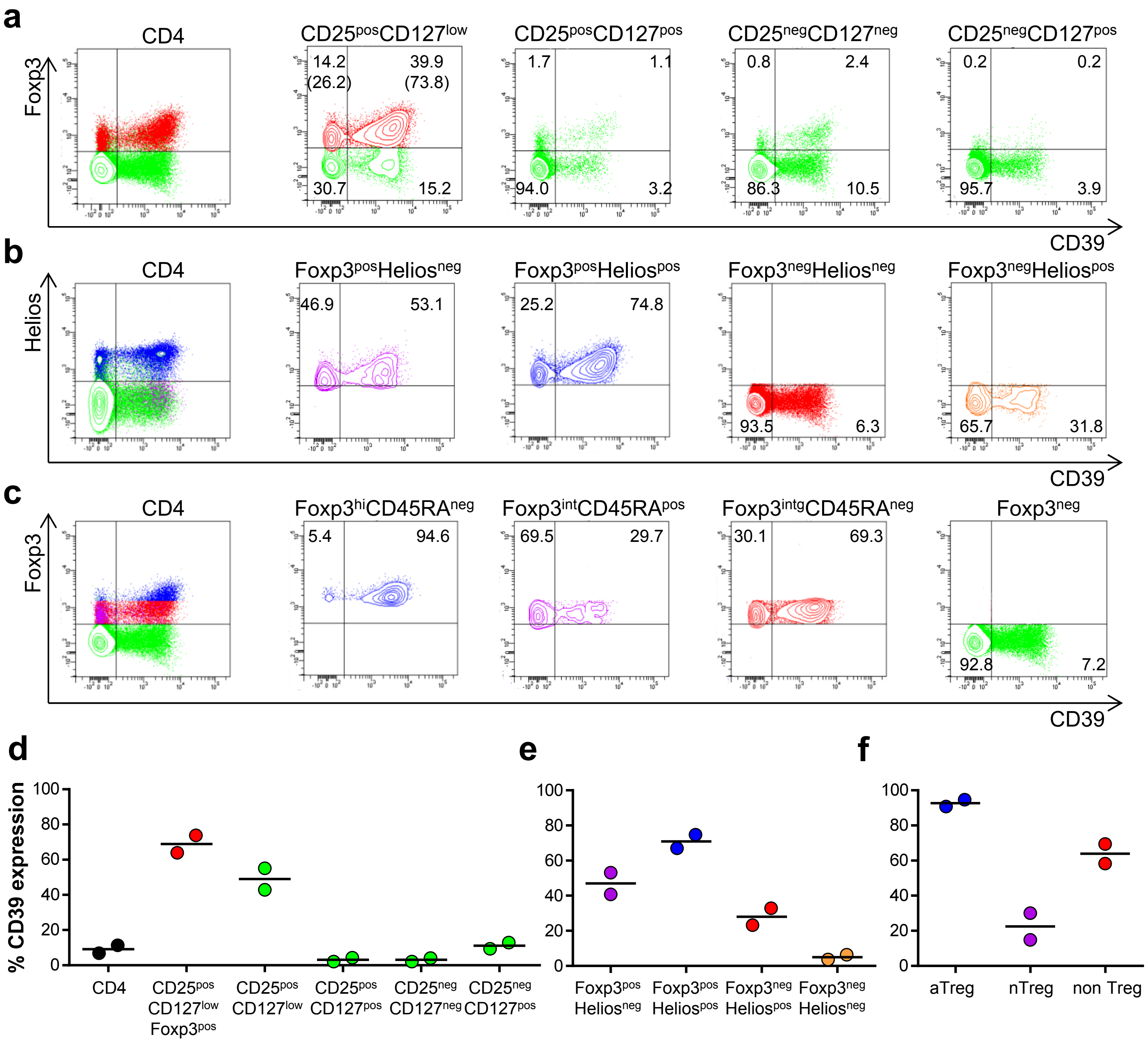

Supplementary figure 7. CD39 analysis. CD39 expression is shown for a representative healthy donor in (**a-c**) and for two HD in (**d-f**). CD39 is given for (Foxp3)CD25CD127 **(a, d)** Foxp3Helios **(b, e)** and Foxp3CD45RA **(c, f)** subsets. Gates were set as described before. Distribution of the designated markers is shown as percentage of cells within the given populations as indicated in the quadrants. Percentage of CD39^{neg} and CD39^{pos} within CD25^{pos} CD127^{low}Foxp3^{pos} def.1 Tregs is given in brackets in **a**.

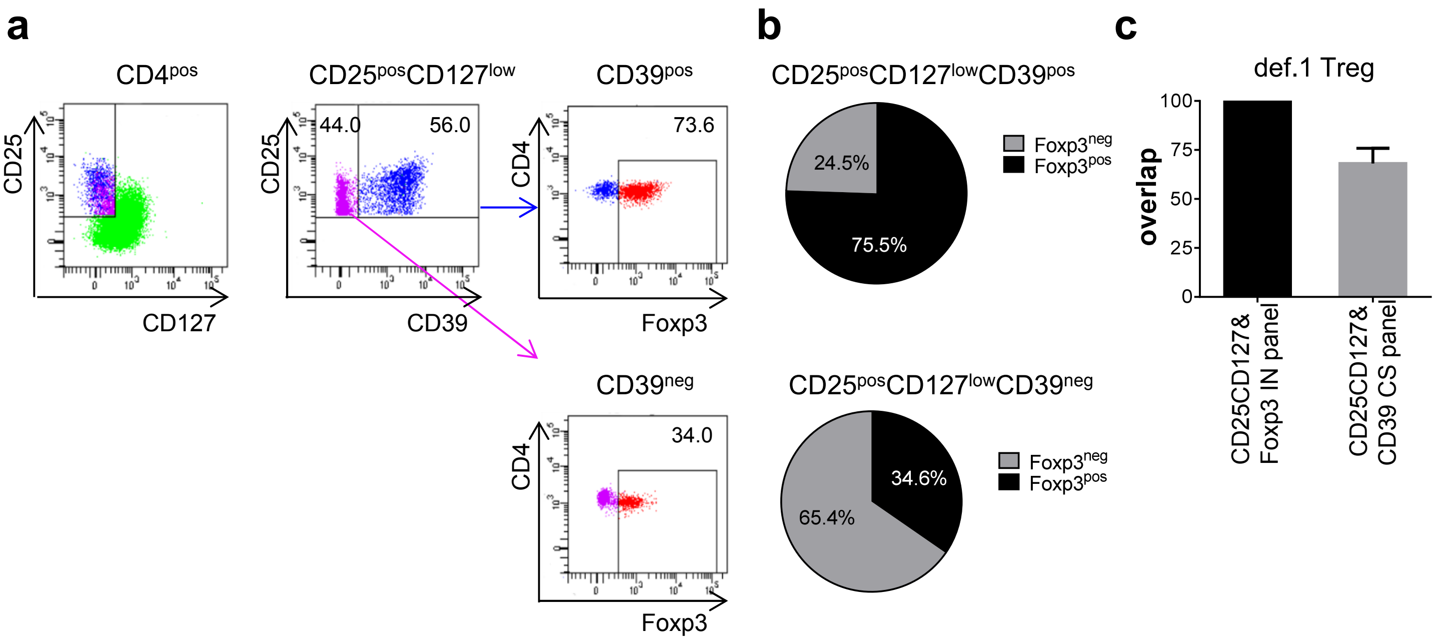

Supplementary figure 8. Def.1 Tregs analysis based on cell surface markers CD25, CD127 and CD39 (and thus omitting intranuclear Foxp3 staining) leads to the detection of less pure def.1 Tregs. PBMC from 2 healthy donors (HD) were analyzed. **a)** FoxP3 expression is depicted in $CD25^{pos}CD127^{low}CD39^{pos}$ and $CD25^{pos}CD127^{low}CD39^{neg}$ cells for a representative patient. Gates for CD25, CD127, CD39 and Foxp3 were set as described before. **b)** Mean percentage FoxP3 expression is depicted within $CD25^{pos}CD127^{low}CD39^{pos}$ and $CD25^{pos}CD127^{low}CD39^{neg}$ cells for two HD. **c)** Overlap between the def.1 Tregs (Foxp3 intranuclear (IN) panel) and FoxP3-positive cells within the $CD25^{pos}CD127^{low}CD39^{pos}$ cells (CD25CD127CD39 cell surface (CS) panel) is depicted. Overlap is calculated in relation to $CD25^{pos}CD127^{low}Foxp3^{pos}$ def.1 Tregs (set at 100) and is given as the mean±SD.

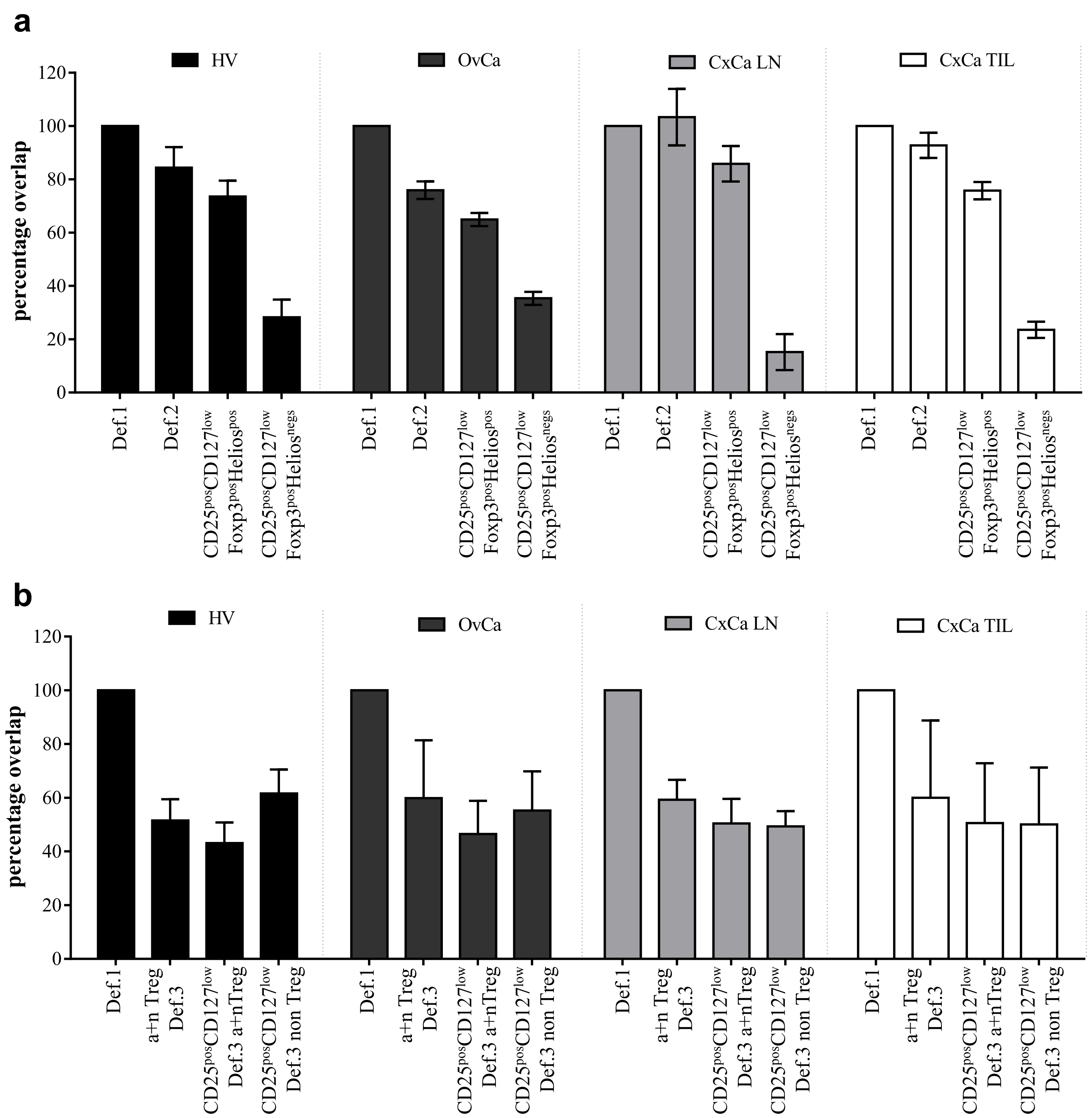

Supplementary figure 9. Analysis of overlap between def.1, def.2 and def.3 Treg subsets for HD- and OvCa patient-derived PBMC, and CxCa-derived TDLN and tumor samples. Overlap between the def.1 and def.2 **(a)** and def.1 and def.3 **(b)** Tregs is given for six PBMC samples from HD and OvCa patients and two tumor draining lymph node (TDLN) and five tumor specimens from CxCa patients. Overlap between the designated populations is calculated in relation to $CD25^{pos}CD127^{low}Foxp3^{pos}$ def.1 Tregs (set at 100; golden standard) and is given as the mean± SD for each population. **a)** Overlap with def. 1 Tregs is analyzed for $Foxp3^{pos}Helios^{pos}$ def.2 Tregs, $CD25^{pos}CD127^{low}$ def.2 Tregs ($Foxp3^{pos}Helios^{pos}$) and $CD25^{pos}CD127^{low}$ $Foxp3^{pos}Helios^{neg}$ def.2 Tregs. **b)** Overlap with def. 1 Tregs is analyzed for a+n def.3 Tregs ($Foxp3^{hi}CD45RA^{neg}$ aTregs + $Foxp3^{int}CD45RA^{pos}$ nTregs), for $CD25^{pos}CD127^{low}$ a+n def.3 Tregs and for $CD25^{pos}CD127^{low}$ non Tregs ($Foxp3^{int}CD45RA^{neg}$ T cells).

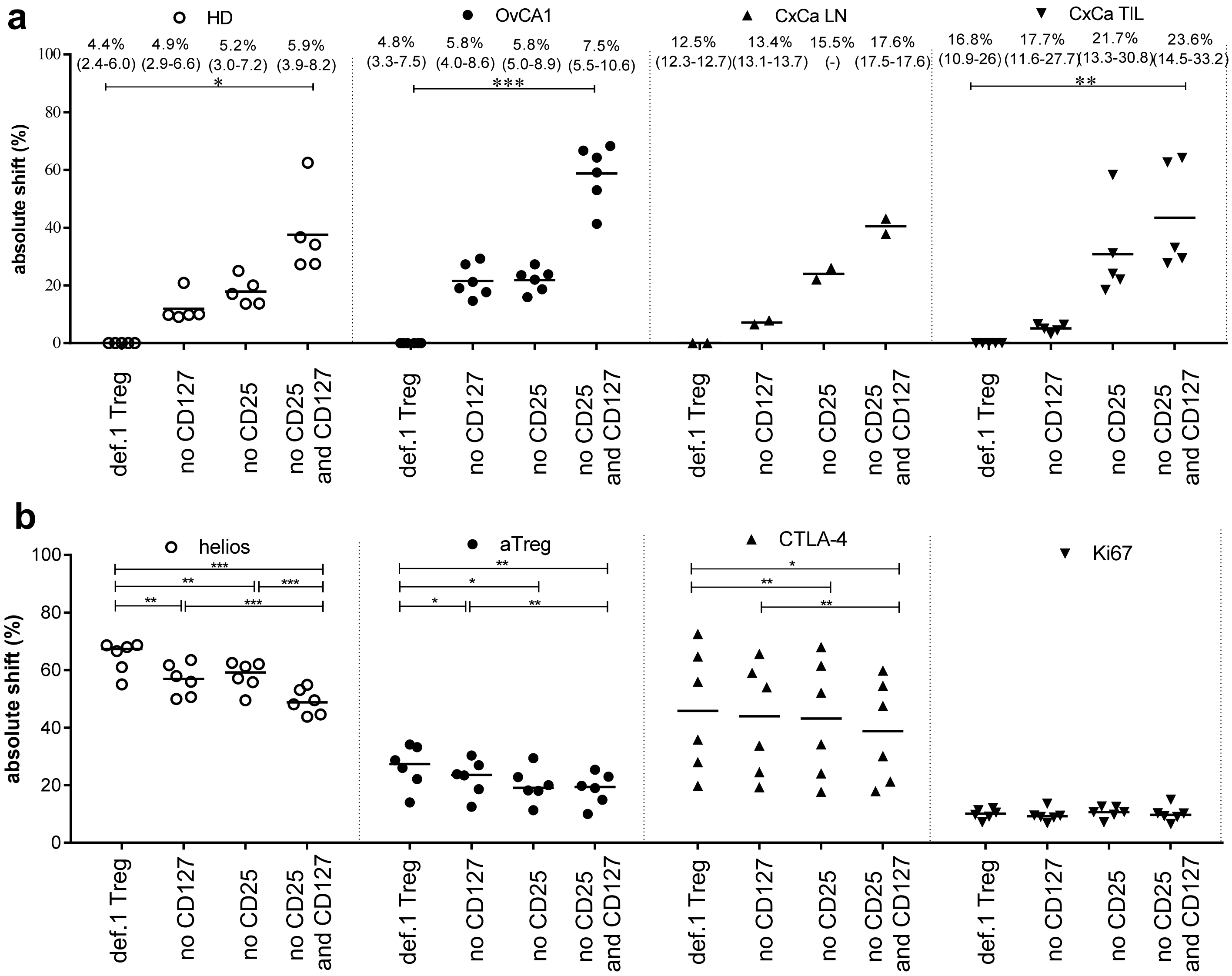

Supplementary figure 10. Further characterization of def.1 Treg analysis in HD- and OvCa patient-derived PBMC, and CxCa-derived TDLN and tumor samples. Removal of CD25 and/or CD127 from def.1 Treg analysis leads to increased frequencies of Tregs. Treg analysis was performed based on CD25 and Foxp3 (no CD127), CD127 and Foxp3 (no CD25), or Foxp3 alone (no CD25 and CD127). Gates for CD25 and CD127 were set as described before. **a)** Tregs were determined by making use of CD25^{pos}CD127^{low}Foxp3^{pos} def.1 and the three adapted def.1 definitions ("no CD127": CD25^{pos}Foxp3^{pos}, "no CD125": CD127^{low}Foxp3^{pos} and "no CD25 and CD127": Foxp3^{pos}) in PBMC of HD and OvCA patients and in TDLN and tumor samples of CxCa patients. Changes in the detected frequencies were given as % shift compared to the CD25^{pos}CD127^{low}Foxp3^{pos} def.1 Treg population. Mean percentage and range of designated population is also given as percentage CD4^{+} T cell for the indicated subsets. **b)** Expression of Helios, Foxp3^{hi}CD45RA^{neg} (i.e. aTregs), CTLA4 and Ki67 were determined within the def.1 Treg and three adapted def.1 Treg populations in PBMC of six OvCa patients. Frequency of helios, Foxp3^{hi}CD45RA^{neg} (i.e. aTregs), CTLA-4 and Ki67-expressing cells are given as percentage of the def.1 and "no CD127" (CD25^{pos}Foxp3^{pos}) , "no CD125" (CD127^{low}Foxp3^{pos}) and "no CD25 and CD127"(Foxp3^{pos}) populations.
